# The Safety and Antiaging Effects of Nicotinamide Mononucleotide in Human Clinical Trials: an Update

**DOI:** 10.1016/j.advnut.2023.08.008

**Published:** 2023-08-22

**Authors:** Qin Song, Xiaofeng Zhou, Kexin Xu, Sishi Liu, Xinqiang Zhu, Jun Yang

**Affiliations:** 1 Department of Occupational and Environmental Health, Hangzhou Normal University School of Public Health, Hangzhou, China; 2 Department of Radiotherapy, The 2^nd^ Affiliated Hospital, Zhejiang University School of Medicine, Hangzhou, China; 3 Department of Nutritional and Toxicological Science, Hangzhou Normal University School of Public Health, Hangzhou, China; 4 Core Facility, The 4^th^ Affiliated Hospital, Zhejiang University School of Medicine, Yiwu, China; 5 Zhejiang Provincial Center for Uterine Cancer Diagnosis and Therapy Research, The Affiliated Women’s Hospital, Zhejiang University School of Medicine, Hangzhou, China

**Keywords:** nicotinamide adenine dinucleotide, nicotinamide mononucleotide, antiaging, clinical trial

## Abstract

The importance of nicotinamide adenine dinucleotide (NAD^+^) in human physiology is well recognized. As the NAD^+^ concentration in human skin, blood, liver, muscle, and brain are thought to decrease with age, finding ways to increase NAD^+^ status could possibly influence the aging process and associated metabolic sequelae. Nicotinamide mononucleotide (NMN) is a precursor for NAD^+^ biosynthesis, and in vitro/in vivo studies have demonstrated that NMN supplementation increases NAD^+^ concentration and could mitigate aging-related disorders such as oxidative stress, DNA damage, neurodegeneration, and inflammatory responses. The promotion of NMN as an antiaging health supplement has gained popularity due to such findings; however, since most studies evaluating the effects of NMN have been conducted in cell or animal models, a concern remains regarding the safety and physiological effects of NMN supplementation in the human population. Nonetheless, a dozen human clinical trials with NMN supplementation are currently underway. This review summarizes the current progress of these trials and NMN/NAD^+^ biology to clarify the potential effects of NMN supplementation and to shed light on future study directions.

**Statement of Significance**

This article integrates the safety and antiaging effects of NMN in preclinical animal studies and human clinical trials, particularly human clinical trials, to clarify the potential benefits of NMN supplementation as well as to shed light on future study directions.

## Introduction

With the decline in the birth rate and extension of life expectancy, the composition of the global population is changing dramatically; in particular, the proportion of citizens over 60 y of age is growing rapidly. For example, in 2019, the global population over 60 was one billion; by 2050, it is expected to reach 2.1 billion, accounting for one-fifth of the world’s population [1]. Aging is a major risk factor for many chronic human diseases; in addition, the incidence of many age-related diseases such as hypertension, atherosclerosis, diabetes mellitus, cancer, Alzheimer’s disease, as well as many other cardiovascular and cerebrovascular diseases have risen drastically, leading to a heavy global socioeconomic and medical burden [[2], [3], [4], [5], [6], [7], [8], [9], [10]]. This phenomenon, combined with the human longing for better health and longer lifespan, has created a huge demand for antiaging (the preventative approach to improve late-life health) products. Among various antiaging healthcare products, as an antiaging product, nicotinamide mononucleotide (NMN) has attracted great attention in North America, Europe, and China over the past decade. NMN is used as a dietary supplement and is widely applied in cosmetic products. The global market for NMN was valued at US $252.7 million in 2020 and is expected to reach US $385.7 million by the end of 2027 [11].

However, on 10 November, 2022, the US Food and Drug Administration (FDA) declared that β-NMN is prohibited as a health supplement. Metro International Biotech LLC had synthesized a proprietary form of β-NMN called MIB-626 that is being developed as a novel drug. Based on the “Federal Food, Drug & Cosmetic Act,” FDA concluded that “NMN was authorized for investigation as a new drug (before being lawfully marketed in supplements) and was the subject of substantial clinical investigations that were instituted and made public.” This finding had the effect of excluding NMN as a dietary supplement. This raised concerns among NMN manufacturers as well as consumers, leading the Natural Products Association and Alliance for Natural Health USA to file a citizen petition with the FDA regarding NMN, and a public hearing was requested in the US Congress to clarify the agency’s position regarding the use of NMN in dietary supplements. How these events will impact NMN’s future consumer market remains to be seen.

NMN is a main precursor of nicotinamide adenine dinucleotide (NAD^+^), an essential coenzyme for vital cellular physiological activities such as metabolism, cell death, aging, DNA repair, gene expression, and neuroinflammation [[12], [13], [14], [15], [16], [17], [18], [19], [20]]. About a century ago, the link between NAD^+^ and health were first established by Conrad Elvehjem, who discovered that pellagra (characterized by dementia, dermatitis, and diarrhea) was caused by a dietary deficiency of niacin (NAD^+^ precursor) in 1937 [21]. As it turns out, NAD^+^, coupled with its reduced form NADH, are key to cellular metabolic processes of all living life forms and have to be maintained in a proper ratio (referred to as the NAD^+^ status). To prevent pellagra, the daily requirement for NAD^+^ synthesis can be achieved by dietary consumption of tryptophan (TRP) or niacin from food. Later on, NAD^+^ depletion is reported to be closely related to aging and several age-related diseases, including various metabolic diseases and cognitive decline [22]. Additionally, studies have shown that NAD^+^ concentrations (in liver and white adipose tissue) decrease under disturbed nutrient conditions [22]. For instance, a high fat or high sugar diet can cause energy overload, ultimately culminating in reduced NAD^+^/NADH ratio (in liver) and decreased NAD^+^ concentrations (in C2C12 myotubes) [23,24]. Also, such diets can lead to increase in blood sugar, insulin levels, and reactive oxygen species formation, which triggers oxidative damage and postprandial oxidative stress [25]. Therefore, it seems that the maintenance of normal NAD^+^ status is important for health, and approaches that can regulate NAD^+^ status, such as nutritional intervention, might be a strategy against aging and metabolic diseases. In fact, it has been shown that caloric restriction increases NAD^+^ bioavailability by activating the expression of NAMPT (nicotinamide phosphoribosyltransferase, which transforms nicotinamide [NAM] to NAD^+^ in the NAD^+^ salvage pathway) [26], whereas it lowers NADH levels and activates sirtuins to extend the life span of yeast [27,28]. Recently, studies have shown that administration of NAD^+^ precursors such as NMN and nicotinamide riboside (NR), which are also present in natural foods, e.g., cow milk, meat, and vegetables, can increase NAD^+^ concentrations in human blood and tissue [29,30], indicating that modulating NAD^+^ metabolism can be a practical target for nutritional intervention. However, it should be kept in mind that although this review discusses NAD^+^ status and concentrations, the terminologies and interpretations are nuanced and complex because *1*) there is no consensus standard as to “high” compared with “adequate” compared with “low” NAD^+^ levels with respect to concentrations that impact cell-specific pathways; and *2*) concentrations may differ by tissue, across species, and even within subcellular pools. Therefore, in many studies, NAD^+^ concentrations were measured in blood or tissue, and such measurements may or may not be an indicator of NAD^+^ status. The only validated surrogate biomarker for NAD^+^ status is urinary markers of NAD^+^ metabolism, such as methylated NAM, which is predictive of pellagra. In contrast, whether blood and/or tissue NAD^+^ concentrations are predictive of disease risk or susceptibility is not yet established (see below). Similarly, whether repletion of blood or tissue NAD^+^ through NMN (or other precursor) supplementation directly modifies the risk of disease, dysfunction, or toxicity in the general population is still to be established. Nonetheless, as an emerging antiaging product in recent years, the impressive results from cell and animal studies [[31], [32], [33], [34]] and those from clinical trials [35,36] are accelerating the market’s growth for NMN supplements. In animal studies, accumulating evidence proved that NMN supplementation could restore NAD^+^ concentrations (in liver, white adipose tissue, skeletal muscle, and primary islets) [24,37] and thus delay the aging process and prevent age-associated diseases. However, clear evidence for antiaging effects (the effects of using preventative approaches to improve late-life health) of NMN on the human body is still scarce. Hence, this review focuses on the research that has assessed NMN’s safety and antiaging effects in human clinical trials.

## The Important Role of NAD^+^ in Aging

NAD exists in 2 forms, the oxidized (NAD^+^) and reduced (NADH) forms, in which NAD^+^ accepts a hydride ion to become NADH. The conversion process is crucial for the central carbon metabolism as NAD^+^ serves as a coenzyme for redox reactions, making it a vital component of energy metabolism [16,30,[38], [39], [40]]; in addition, it is an essential cofactor for nonredox enzymes such as sirtuins and poly(adenosine diphosphate-ribose) polymerases (PARPs) [12,22,41]. It is also critical for maintaining tissue and metabolic homeostasis for healthy aging. There have been extensive reviews of the relationship between NAD^+^ and the 9 aging hallmarks, namely genomic instability [[42], [43], [44], [45]], telomere attrition [46], epigenetic alterations [47], loss of proteostasis [44,[48], [49], [50]], deregulated nutrient sensing [[51], [52], [53]], mitochondrial dysfunction [54,55], cellular senescence [55,56], stem cell exhaustion [[57], [58], [59], [60]], and altered intercellular communication [[60], [61], [62], [63]]. Aging is accompanied by a gradual decline of NAD^+^ concentration across multiple human tissues, including skin, blood, liver, muscle, and brain [[64], [65], [66], [67], [68], [69], [70]]. For instance, the average NAD^+^ concentration in human skin tissues is several times lower in adults than in newborn babies [66]. Two magnetic resonance imaging-based studies revealed that NAD^+^ concentrations in the human brain declined 10% to 25% from young adulthood to old age [69,70]. Many factors, including DNA damage, chronic inflammation, oxidative stress [31], and increased NAD^+^-consuming enzyme activities [71], have also been shown to accelerate NAD^+^ degradation. Lowering the concentrations of NAD^+^ in cell or tissue results in decreased energy production within mitochondria, which contributes to the development of aging and a range of age-related disorders, including atherosclerosis, arthritis, hypertension, cognitive decline, diabetes, and cancer [22,[72], [73], [74], [75], [76], [77], [78]].

**FIGURE 1:**
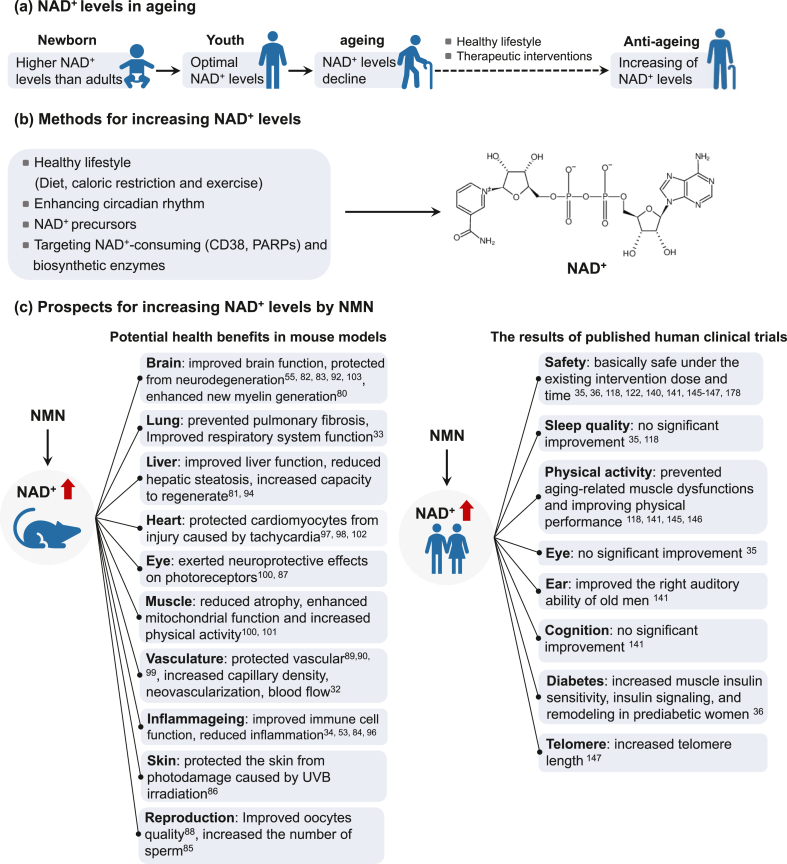
The changes of NAD^+^ levels during aging (a), the approaches to restore NAD^+^ levels (b), and the health benefits of restoring NAD^+^ levels by NMN supplementation (c). NAD, nicotinamide adenine dinucleotide; NMN, nicotinamide mononucleotide.

NAD^+^-dependent mechanisms in aging have been summarized by Covarrubias et al. [22] and McReynolds et al. [79] as follows: *1*) Metabolic dysfunction. The relationship between NAD^+^ and metabolism has been known for almost a century. Changing or disrupting metabolic status caused by factors such as a high-fat diet, postpartum weight loss, and disruption of the circadian rhythm can lead to lower NAD^+^ concentrations (in the liver and white adipose tissue), thus reducing the activity of sirtuins and other NAD^+^-dependent cellular processes; *2*) Inflammation. Numerous studies have showcased the regulatory function of NAD^+^ and NAD^+^-consuming enzymes in the biology of macrophages, T cells, and B cells. With aging being a known factor and a significant catalyst for numerous diseases, heightened expression of proinflammatory cytokines can cause increased inflammation, leading to tissue and DNA damage and further activation of major NAD^+^-consuming enzymes, such as CD38 and PARPs, resulting in a hastened decline of NAD^+^ in macrophages; *3*) Senescence. The senescence-associated secretory phenotype (SASP) of aging cells depends on the NAD^+^ concentration in senescent cells. In addition, CD38 levels are increased in aging tissues, which could at least partially explain the age-related decrease in liver NAD^+^ concentration; *4*) Neurodegeneration, which is linked to a decline in NAD^+^ concentrations in the brain, is closely associated with aging and various neurodegenerative disorders. Axonal degeneration, a forerunner to many neuronal diseases associated with aging, is identified by swift NAD^+^ depletion, which is attributed to a reduction in the NAD^+^ biosynthetic enzyme nicotinamide mononucleotide adenylyltransferase 2 (NMNAT2). Furthermore, the NAD^+^-consuming enzyme SARM1 is activated by axonal injury and mediates axonal degeneration by promoting NAD^+^ degradation. Therefore, strategies to increase NAD^+^ are of great interest in antiaging and longevity studies. Indeed, preclinical studies of NMN intervention in mouse models have shown that increasing NAD^+^ concentration could prevent and treat age-related diseases by improving tissue and organ function, reducing inflammation, enhancing immune and reproductive function, increasing physiological benefits, and protecting cognitive function (Table 1). These benefits may work together to improve health and perhaps increase lifespan. Several potential strategies are available to boost NAD^+^ concentrations (in blood, myocardial cell, adipose tissue, liver, brain, or U2OS cells), including lifestyle changes (such as exercise [105,106], diet and caloric restriction [107,108], and enhancing circadian rhythm [109,110]), use of small-molecule inhibitors or activators to boost NAD^+^ biosynthesis [72,[111], [112], [113], [114], [115], [116]],and supplementation with NAD^+^ precursors (primarily NMN, NR, and NAM) (Figure 1) [22]. Supplementation with NAD^+^ precursors and activation of NAD^+^ biosynthetic enzymes/inhibition of NAD^+^ degradation have produced health benefits in mouse models. However, only NAD^+^ [117] and NAD^+^ precursors (NA, NMN, NR, and NAM) are being explored in humans [29,[118], [119], [120], [121], [122]].

**TABLE 1:** Preclinical studies (after 2015) in mouse models using NAD^+^ boosting strategies (NMN intervention)

Model	NMN dose	Potential health benefits	References
C57BL/6, G3 Terc^−/−^, SIRT2^−/−^, Sirt2^flox/flox^ and NG2-Cre^ERT34^ mice	Intraperitoneal injection: 10 mg/kg body weight	Restored nuclear entry of Sirt2 and rejuvenated aged oligodendrocyte progenitor cells; Enhanced new myelin generation in aged central nervous system	[80]
C57BL/6 wild-type mice and Sirt3-deficient mouse	Intraperitoneal injection: 500 mg/kg body weight	Improved stress resistance against acetaminophen-induced liver injury, restored Nrf2-mediated adaptive homeostasis; Restored liver redox homeostasis via the Sirt3–Nrf2 axis and protected aged liver from oxidative stress-induced injury	[81]
Aged 4 wk male C57BL/6J mice	Oral administration: 400 mg/kg body weight	Increased brain NAD^+^ levels in mice after 45 min oral intervention	[82]
Middle cerebral artery occlusion mouse	Intraperitoneal injection: 300 and 2000 mg/kg body weight	NMN accumulated earlier than NAD in the brain, reduced cerebral infarction at 24 h post-middle cerebral artery occlusion; Protected from acute ischemic stroke injury	[83]
Aged 5–6 wk male Kunming mice	Intraperitoneal injection: 300, 400 and 500 mg/kg body weight	Modulated GABA and glutamate production by increasing GABA_A_ receptor α2 and glutamic acid decarboxylase 65/67 expression; Enhanced immune system by boosting nitric oxide secretion and IL-1β expression	[84]
STZ-induced diabetic C57BL/6J mice	Oral administration: 500 mg/kg body weight	Significantly increased body and testis weight and number of sperm in STZ-induced diabetic mice	[85]
Aged (16 mo) male C57BL/6J mice	Intraperitoneal injection: 500 mg/L	Improved the intestinal structural and functional decline; the potential mechanism was boosting the NAD^+^ pool and activating the SIRT3/6-mediated signaling pathway with regard to antioxidant, anti-inflammatory, and barrier function	[34]
C57BL/6 mice	Oral administration: 500 mg/kg body weight	Prevented lung physiological decline and pulmonary fibrosis; Improved respiratory system function	[33]
Aged 7 wk female ICR mice	Intraperitoneal injection: 250 mg/kg body weight	Blocked UVB-induced photodamage in mice, maintaining normal structure and amount of collagen fibers, normal thickness of epidermis and dermis, reducing the production of mast cells, and maintaining complete organized skin structure	[86]
Aged (7–10 wk) C57BL/6 mice	Intraperitoneal injection: 250 and 500 mg/kg body weight	Increased NAD^+^ levels, SIRT1 protein expression, and heme oxygenase-1 expression; Exerted neuroprotective effects on photoreceptors after retinal detachment and oxidative injury	[87]
ICR mice	Intraperitoneal injection: 200 mg/kg body weight	Improved the quality of oocytes from naturally aged mice by recovering NAD^+^ levels; Increased ovulation of aged oocytes but also enhanced their meiotic competency and fertilization ability by maintaining the normal spindle/chromosome structure and the dynamics of the cortical granule component ovastacin	[88]
Aged 3 and 24 mo male C57BL/6J mice	Intraperitoneal injection: 500 mg/kg body weight	Protected vascular system by changing miRNA expression profile	[89]
Aged 3 and 24 mo male C57BL/6 mice	Intraperitoneal injection: 500 mg/kg body weight	Restored youthful expression levels in 204 genes; Promoted SIRT1 activation in the neurovascular unit; Protected neurovascular function;	[90]
Aged 3 wk male Sprague Dawley rat	Intraperitoneal injection: 20 mg/kg body weight	Alleviated Al-induced bone injuries by decreasing bone loss, suppressed oxidative stress as well as inhibited thioredoxin-interacting protein-NOD-like receptor pyrin domain containing 3 inflammasome pathway and proinflammatory cytokine production	[91]
Aged (24 and 3 mo) male Wistar rats	Intraperitoneal injection: 100 mg/kg body weight	Reversed aging-induced learning and memory impairment; Improved mitochondrial function in the brains of aged animals; Reduced apoptosis in the brains of aged animals	[92]
Aged (18 mo) C57BL/6J mice	Oral administration: 400 mg/kg body weight	Increased endurance; Improved blood flow in elderly mice by increasing capillary density	[32]
Aged (24 mo) C57BL/6 male mice	Intraperitoneal injection: 500 mg/kg body weight	Reversed aging-induced cerebrovascular endothelial dysfunction; Restored NAD^+^ and mitochondrial energetics and reduced mtROS; Improved cognitive performance in NMN treated aged mice	[93]
Male Long-Evans rats (decompensated hemorrhagic model)	Oral administration: 400 mg/kg body weight	Reduced lactic acidosis and serum IL-6 levels, increased NAD^+^ levels, and prevented mitochondrial dysfunction in both liver and kidney; Mitigated inflammation, improved cellular metabolism, and promoted survival following hemorrhagic shock	[94]
Aged (6 mo old) APP_(swe)_/PS1_(DE9)_ double transgenic (Alzheimer disease model) mice	Subcutaneous injection: 100 mg/kg body weight for 28 d	Reduced inflammatory responses, synaptic loss, amyloid plaque burden and β-amyloid production by inhibition of JNK activation	[53]
Middle cerebral artery occlusion CD1 mice	Intraperitoneal injection: 300 mg/kg body weight	Decreased mortality, brain infarction, edema, apoptosis, and hemorrhage via protecting blood–brain-barrier integrity	[95]
Collagenase-induced intracerebral hemorrhage CD1 mice	Intraperitoneal injection: 300 mg/kg body weight	Reduced brain edema, brain cell death, oxidative stress, neuroinflammation, intercellular adhesion molecule-1 expression, microglia activation and neutrophil infiltration in brain hemorrhagic area by suppressing neuroinflammation/oxidative stress	[96]
Male cardiac-specific FXN-knockout mice and male SIRT3-knockout/FKN-knockout mice	Intraperitoneal injection: 500 mg/kg body weight	Improved cardiac functions, reduced energy waste and improved energy utilization in FXN-knockout mice but not in SIRT3-knockout/FKN-knockout mice	[97]
Cardiac-specific deficiency of Klf4 C57BL/6J mice	Intraperitoneal injection: 500 mg/kg body weight for 3 or 5 d	Preserved mitochondrial ultrastructure, reduced ROS, and prevented cell death in the heart; Protected the mutant mice from pressure overload-induced heart failure	[98]
Aged (26–28 mo) C57BI/6 male mice	Oral administration: 300 mg/kg body weight	Restored SIRT1 activity and reversed age-related arterial dysfunction by decreasing oxidative stress	[99]
C57BL/6N male mice	Oral administration: 100 and 300 mg/kg body weight	Suppressed body weight gain; Improved eye function, healthy plasma lipid profile, insulin sensitivity, physical activity, energy metabolism, and other physiopathologies; Enhanced mitonuclear protein imbalance and mitochondrial oxidative metabolism in skeletal muscles	[100]
High-fat diet-fed aged C57BL6/J female mice	Intraperitoneal injection: 500 mg/kg body weight	Increased liver citrate synthase activity and triglyceride accumulation; Improved glucose tolerance, NAD^+^ levels of muscle and liver	[101]
Transverse aortic constriction-stressed mice, male conditional knockout mice	Intraperitoneal injection: 500 mg/kg body weight	Improved mitochondrial function and protected mice from heart failure	[102]
Aged 3 mo osteoporotic male C57BL/6 mice	Oral administration: 31.25, 62.5, 125, 250 and 500 mg/kg body weight	Dramatically ameliorated the hippocampal CA1 injury and significantly improved neurological outcome; Prevented the increase in PAR formation and NAD^+^ catabolism	[103]
Male Wistar rats (Alzheimer disease model)	Intraperitoneal injection: 500 mg/kg body weight	Improved cognitive function and energy metabolism, ameliorated neuron survival, reduced ROS accumulation	[55]
APP_(swe)_/PS1_(DE9)_ double transgenic (Alzheimer disease model) mice	Subcutaneous injection: 100 mg/kg body weight	Decreased brain APP levels and increases brain mitochondrial function; Reversed cognitive deficits	[104]

Al, aluminum; APP, amyloid precursor protein; FKN, fractalkine; FXN, frataxin; GABA, γ-aminobutyric acid; IL, interleukin miRNA, microRNA; mtROS, mitochondrial reactive oxygen species; NAD, nicotinamide adenine dinucleotide; NMN, nicotinamide mononucleotide; NOD, nucleotide-binding oligomerization domain; Nrf, nuclear factor erythroid 2-related factor; PAR, poly-ADP-ribose; ROS, reactive oxygen species; SIRT, sirtuin; STZ, streptozotocin; UVB, ultraviolet B;

Numerous studies have validated that alterations in NAD^+^ homeostasis can adversely impact the normal functions of cells. Nevertheless, a precise definition of the association between NAD^+^ homeostasis and human health outcomes remains necessary. Recently, Zapata et al. [123] reviewed the relationship between NAD^+^ homeostasis and human health outcomes. They highlighted that NAD^+^ depletion could lead to various pathological phenotypes, including rare inherited defects, Leber congenital amaurosis, severe neonatal encephalopathy, and pellagra [123]. Primary NAD^+^ deficiencies result from impaired biosynthesis, such as when deleterious variants of NAD^+^-related genes are mutated. In contrast, the secondary deficiencies may be caused by other factors affecting NAD^+^ homeostasis, such as increased NAD^+^ consumption or a dietary deficiency of NAD^+^ precursors [123]. Furthermore, several recent epidemiological studies have attempted to define the relationship between NAD^+^ concentrations (in blood, sperm, and skeletal muscle) and disease. Tran et al. [124] evaluated skeletal muscle NAD^+^ and NADH concentrations in asymptomatic middle-aged people with HIV, revealing that decreased NAD^+^ concentrations in skeletal muscle are related to increased physiological weakness and coinfection with the virus. Yang et al. [125] analyzed the relationship between blood NAD^+^ concentrations and anemia in 727 female participants from the Jidong community in China. Blood samples were collected from the large antecubital veins after overnight fasting. NAD^+^ concentrations in blood were then stratified into 4 categories: Q1 (<27.6 μmol), Q2 (27.6–31.0 μmol), Q3 (31.0–34.5 μmol), and Q4 (≥34.5 μmol). The study findings indicated that an increased concentration of blood NAD^+^ was strongly linked to a decreased occurrence of anemia among women, specifically microcytic and normocytic anemia [125]. Bai et al. [126] found that sperm NAD^+^ concentration was independent of age and negatively correlated with sperm quality in males, indicating that NAD^+^ has a unique role in spermatogenesis. Xiao et al. [127] analyzed the metabolomics and cytokine/chemokine profiling in serum samples from 17 healthy controls and 20 mild and 44 severe COVID-19 patients and observed that NAD^+^ concentrations decreased with the increase in the severity of COVID-19. However, such observations remain correlational and do not prove the relationship between NAD^+^ and diseases. Furthermore, it remains to be determined how much NAD^+^ is required for normal tissue function and the threshold level to trigger pathophysiological changes in different tissues.

### NAD^+^ Biosynthesis and NAD^+^ Precursors

**FIGURE 2:**
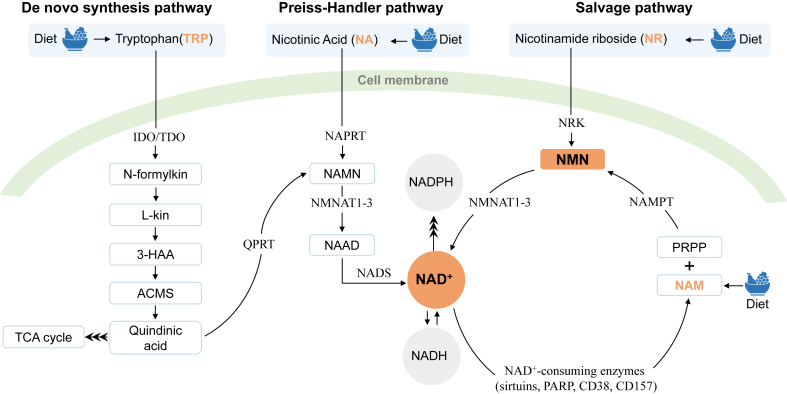
NAD^+^ levels are maintained by 3 independent pathways. The salvage pathway is the major source of NAD^+^. 3-HAA, 3-hydroxyanthranilic acid; ACMS, 2-amino-3-carboxymuconate-6- semialdehyde; cADPR, cyclic ADP-ribose; IDO, indoleamine 2,3-dioxygenase; L-kin, L-kinurenine; NAAD, nicotinic acid adenine dinucleotide; NAD, nicotinamide adenine dinucleotide; NADS, NAD^+^ synthase; NAM; nicotinamide; NAMN, nicotinic acid mononucleotide; NAMPT, nicotinamide phosphoribosyltransferase; NAPRT, nicotinic acid phosphoribosyltransferase; NMNAT, nicotinamide mononucleotide adenylyltransferase; NRK, nicotinamide riboside kinase; PARP, poly(adenosine diphosphate-ribose) polymerase; PRPP, 5-phosphoribosyl-1-pyrophosphate; TDO, tryptophan 2,3-dioxygenase.

NAD^+^ can be synthesized from diverse dietary sources, including TRP, nicotinic acid (NA), NR, and NAM. The NAD^+^ biosynthesis pathways include the de novo synthesis pathway, Preiss-Handler pathway, and salvage pathway [12], as illustrated in Figure 2. In the de novo synthesis pathway, TRP goes through a series of reactions in 8 steps to generate NAD^+^. TRP, as an NAD^+^ precursor, is first converted to quinolinic acid (QA) through a 5-step enzymatic reaction and then to nicotinic acid mononucleotide (NAMN) under the action of quinolinic acid phosphoribosyl transferase (QPRT). In addition, QA can also enter the tricarboxylic acid cycle. QPRT is the most critical rate-limiting enzyme in the de novo synthesis pathway. The enzyme catalyzes the reaction step in an ATP-dependent manner that requires the participation of Mg^2+^ and 5-phosphoribosyl-1-pyrophosphate (PRPP). Finally, NAMN enters the Preiss-Handler pathway.

In the Preiss-Handler pathway, NA is catalyzed by nicotinic acid phosphoribosyl transferase to generate NAMN. Subsequently, NAMN is catalyzed by NMNAT to generate nicotinic acid adenine dinucleotide (NAAD). Afterward, NAAD is converted to NAD^+^ under NAD^+^ synthetase.

Among the 3 pathways, the de novo biosynthetic pathway is the most indirect mechanism contributing to system-wide NAD^+^, with most NAD^+^ coming from the NAM salvage pathway [64,128]. In the salvage pathway, NAM, as an NAD^+^ precursor, comes from diverse dietary sources and by-products of NAD^+^-consuming enzymes such as NAD^+^-dependent protein deacetylase (sirtuin), PARP, and CD38. First, NAM is catalyzed by NAMPT to generate NMN, and then NMN is catalyzed by NMNAT to generate NAD^+^. As a precursor of NAD^+^, NR can generate NAD^+^ under the action of nicotinamide riboside kinases in the salvage pathway.

The metabolic pathways of NAD^+^ precursors can facilitate their conversion into NAD^+^ within the human body. Yiasemides et al. [129] reported that oral administration of 500 mg NAM for 1 h significantly increased human blood NAD^+^ concentration 1.3-fold and significantly reduced UV immunosuppression in the skin. Oral administration of 1000 mg/d NR for 1, 3 and 6 wk significantly increased NAD^+^ concentration 2.7-fold [130] in peripheral blood mononuclear cells (PBMCs) of a 52-y-old healthy participant, 2.3-fold [131] in the whole blood of older participants (average age of 75), and 1.7-fold [120] in PBMCs of healthy participants (55–79 y). In addition, the concentration of NAD^+^ metabolites significantly increased in the blood, skeletal muscle, and urine [120,130,131]. Other NR human clinical trials also showed that NR could significantly increase the concentrations of NAD^+^ by about 1- to 2-fold [[132], [133], [134], [135], [136], [137]] and NAD^+^ metabolites in the blood [134,[137], [138], [139]]. Oral administration of 250 mg/d NMN for 4, 8, and 12 wk also significantly increased baseline NAD^+^ concentration 2.5-fold, 2-fold, and 1.7-fold, respectively, in the whole blood of healthy participants, simultaneously raising NAD^+^ metabolite concentrations in the whole blood and skeletal muscle of healthy participants [140]. No significant changes in the baseline concentrations of NAD^+^ and its metabolites in whole human blood were observed even after 16 wk of treatment with the same dose of NMN [140]. Furthermore, Igarashi et al. [141] found that oral administration of 250 mg/d NMN for 12 wk significantly increased baseline NAD^+^ concentration by 2.57-fold in whole blood. NAD^+^ metabolite concentrations in the whole blood of healthy participants and muscle strength and performance were also significantly improved [141]. Oral administration of 250 mg/d NMN for 10 wk could significantly increase the baseline NAD^+^ concentration in PBMCs of prediabetic women and increase muscle insulin sensitivity but not the NAD^+^ concentration in skeletal muscle [36]. Similarly, in NR human clinical trials, NAD^+^ concentrations also were not increased in skeletal muscle [131,139,142]. These details are summarized in Supplementary Table 1, which shows the effects of different NAD^+^ precursors on NAD^+^ and its metabolites in human clinical trials [35,36,119,120,122,[129], [130], [131], [132], [133], [134], [135], [136], [137], [138], [139], [140], [141], [142], [143], [144]). The above human clinical trials confirmed that these NAD^+^ precursors could improve NAD^+^ status in the blood; however, the bioavailability, quantitative metabolomics, or pharmacokinetics of NA, NAM, NR, and NMN have yet to be systematically compared. Recently, a clinical trial (NCT05517122) entitled “Effect of oral NAD^+^ precursors (NAM, NR, NMN) administration on blood NAD^+^ concentration in healthy adults” was launched, and its results are pending.

Most of the preclinical models and clinical studies published to date demonstrate NAD^+^ precursor supplementation as a means of remedying tissue dysfunction linked to severely compromised NAD^+^ metabolism. However, whether maintenance of normal NAD^+^ status through diet or NAD^+^ precursor supplementation can delay age-related functional decline and reduce risk of disease in the general population remains to be clearly established. Several NMN clinical trials were conducted on healthy volunteers, and the results showed that NMN increased aerobic capacity during exercise training [145], improved muscle strength and performance during the 30-second chair stand test and walking speed [141], decreased blood pressure, pulse pressure, and blood glucose [146], and increased the telomere length of PBMCs [147]. On the whole, it is simply too early to state with confidence whether NAD^+^ supplementation will delay age-related functional decline and reduce the risk of disease in the general population because longer-term studies with large populations have not been performed.

The primary precursors for NAD^+^ are NA, NAM, NR, and NMN. NA acts like a vitamin in preventing pellagra and is a widely used drug for dyslipidemia treatment. Its pharmacological effect involves inhibiting adipose tissue lipolysis, leading to a reduction in free fatty acid concentration and its transport to the liver [148]. Compared with other precursors, the side effects of NA are well known. The most common adverse effects of NA include flushing, rash, hyperglycemia, hyperuricemia, and gastrointestinal disorders. Additional side effects of NA have also been reported, including a small reduction in both platelet count and prolongation of prothrombin time, rhabdomyolysis, and other dermopathies [148]. NAM is a water-soluble compound found in meat, fish, mushrooms, grains, nuts, and legumes [149]. Besides treating pellagra, NAM has a potential for clinical use in the prevention and treatment of various diseases, such as rosacea, acne [149], and hyperphosphatemia [150]. Furthermore, NAM has been tested for the treatment of diabetes. The European NAM diabetes intervention test allowed children to receive 5 to 3000 mg NAM daily for 5 y. No significant difference in the incidence rate of adverse events was found between the NAM and placebo groups [151]. Supplementing 1 g of NAM daily is a safe and effective way to improve the metabolic abnormalities and quality of life in diabetes patients with nonalcoholic fatty liver disease [152]. Compared to NA, NAM has shown far greater tolerability in humans, and its tolerance dose can be almost up to 3 g/d in adults [149]. Nonetheless, certain side effects exist, including epigenetic changes, impeded bioenergetics and gastrointestinal disturbance (nausea, vomiting, diarrhea) for NAM [149,153]. Moreover, NAM has shorter retention in rats’ bodies than NMN [154]. On the other hand, existing human clinical trials have not shown any side effects of NR and NMN. While the pharmacokinetics and metabolic mechanisms of NR and NMN are still being investigated, it is important to note that not all cells have the ability to convert all NAD^+^ precursors to NAD^+^ [155]. The fates of NAD^+^ precursors appear to depend on the tissue distribution and expression levels of NAD^+^ biosynthetic enzymes, nucleosidase, and presumptive transporters for each specific precursor. Furthermore, these precursors are differentially utilized in tissues and organs [155,156]. To identify the potential mechanisms for the physiological and side effects of each precursor, it is necessary to comprehend the distinct features of the metabolism of every NAD^+^ precursor.

Interestingly, as a fact that existed in the antiaging market before the FDA ban was executed, NMN was the dominating product in the antiaging consumer market. For example, a search on Amazon.com for ‘NMN’ returned 6 pages (each page contains 60 listings) of various brands of NMN products; after excluding some duplicated or inaccurate listings, the total number is still close to 300. In contrast, a search for ‘NA’ showed around 40 brands, ‘NAM’ with ∼20 and ‘NR’ with only 4 brands (search conducted on 26 January, 2023). Theoretically, both NR and NMN can increase NAD^+^ concentrations, making them equally competitive in the consumer market. However, from a biochemist’s perspective, NR holds an edge over NMN because cells cannot directly absorb NMN, and NMN must be converted to NR before entering cells. Thus, this market anomaly may be attributed to the fact that NMN is a more direct precursor in the biosynthesis pathway of NAD^+^ when viewed from a layman’s standpoint (Figure 2). This illusion, probably further fanned by the manufacturers, is responsible for the hyped demands and sales of NMN to consumers that causes more people to be exposed to NMN compared to other NAD^+^ precursors. Consequently, NMN should receive more attention for its effects and safety issues. For more information about NR, NAM, and NA, we refer the readers to several reviews dealing with this topic [29,155,157,158]. In this review, we only focus on NMN.

### NMN as NAD^+^ Precursor

Since NAD^+^ cannot be absorbed orally or pass through the cell membrane, elevating NAD^+^ concentrations can be accomplished by supplementing the diet with its precursors, which include TRP, NA, NAM, NR, and NMN. To prevent pellagra resulting from a lack of tryptophan, NA and NAM dietary supplements have been employed. Nonetheless, due to their potential side effects, such as flushing and inhibiting PARPs and sirtuins, both NA and NAM should be used with caution [159]. Therefore, NMN and NR have gained priority as agents to raise NAD^+^ concentrations. As explained above, compared with NR, NMN is a more direct precursor of NAD^+^. Unfortunately, all the dietary precursors of NAD^+^, including TRP, NA, NR, and NAM, are imported directly into the cells and can produce NAD^+^, except NMN. Although studies have shown that NMN can increase NAD^+^ concentrations in rodents and humans, how NMN is absorbed by cells and tissue is still controversial and needs to be better understood. Two mechanisms have been proposed to explain NMN uptake in cells or tissues. One is that the cell can directly absorb NMN through a specific transporter, Slc12a8, a cell membrane transporter in the aged mouse ileum [160]. However, this observation had some challenges [161,162]. Furthermore, it is necessary to verify the expression profile of Slc12a8 in the human gastrointestinal tract. Another more widely accepted mechanism entails the dephosphorylation of extracellular NMN into NR by ectonucleotidases (e.g., CD73) prior to its cellular absorption [[162], [163], [164], [165]]. After NR enters the cell through equilibrative nucleoside transporters (ENT1, ENT2, and ENT4), it is phosphorylated by NRK1 and NRK2 to generate NMN [162,163,166,167]. This mechanism is also supported by the results of Kim et al. [167], who showed via isotope-labeled NMN that most orally ingested NMN was converted to NR in intestinal tissue. The study suggested that the NMN uptake bypasses direct transport and is first dephosphorylated to NR to promote intestinal absorption [168].

NMN is a bioactive nucleotide with a molecular weight of 334.22 g/mol and has α and β configurations, with the β configuration showing better bioactivity. It has good water solubility, and its solution is acidic. In the human body, NMN can be found in placental tissue, blood, urine, and other bodily fluids [100] and is mainly distributed in the nucleus, mitochondria, and cytoplasm of cells [169]. NMN occurs naturally in an assortment of vegetables, fruits, and meats, such as cabbage, tomato, mushroom, soybean, pear, orange, cherry, shrimp, scallops, beef, and salmon. The concentration of NMN varies across these food items, ranging from 0.035 to 1.88 mg/100 g in vegetables, 0.021 to 1.6 mg/100 g in fruits, and 0.029 to 0.51 mg/100 g in aquatic products [100,167]. The most studied NAD^+^ precursors in humans are NA and NAM, collectively termed niacin and/or vitamin B_3_. The most fundamental use of NAM and NA is to protect against pellagra, which can develop from TRP deficiency [29,167,170]. Dietary TRP is also classified as a niacin equivalent, and 60 mg of TRP is considered the equivalent of 1 mg of niacin [170]. The recommended daily allowance (RDA) of NA is 16 and 14 mgNE/d for adult men and women, respectively [170]. However, an excessive human oral dose of NAM and NA can cause side effects, including vasodilation (30–100 mg/d NA) [171], headache, hypotension, liver toxicity [172,173], glucose intolerance [174], epigenetic changes, impeded bioenergetics, potential carcinogenesis, blurred vision (1.5 g/d NA) [175], and gastrointestinal tract disturbances (nausea, vomiting, diarrhea) (3 g/d NAM) [174,176]. NR is found in trace amounts in cow’s milk [177] and has beneficial effects in multiple conditions in rodents and humans. Clinical studies have demonstrated the safety of NR, with oral doses ranging from 100 to 2000 mg/d in human trials, as displayed in Supplementary Table 1. It should be noted, however, that overconsumption of any substance can lead to toxicity. So far, existing human clinical trials have not shown any side effects of NR and NMN (Table 2 and Supplementary Table 1). Neither NMN nor NR has an established official RDA.

**TABLE 2:** The safety and antiaging effects of NMN in human clinical trials

Registration number	Design	Dose & duration	Indicators	Outcome	Location	References
UMIN000021309	Nonblinded, nonrandomized, non–placebo-controlled study; 10 healthy men aged 40–60 y	Oral administration: 100, 250 or 500 mg for 5 h	Clinical parameters, ophthalmic parameters, sleep quality score, serum parameters, NMN metabolites levels in plasma	↑NMN metabolites (2Py and 4Py) in plasma and bilirubin levels; ↓creatinine, chloride, and glucose levels within the normal ranges in serum; No significant changes in ophthalmic examination and sleep quality score; Single oral administration of NMN up to 500 mg is safe and well-tolerated in healthy men without causing any significant deleterious effects	Japan	[35]
jRCTs041200034	Double-blind, randomized, placebo-controlled study; 30 healthy volunteers aged 20–65 y	Oral administration: 250 mg daily for 12 wk	Adverse events, clinical parameters, blood and urine biochemical parameters, body composition, skeletal muscle mass, bone mineral mass, NAD^+^, and amino acid metabolome of blood	↑NAD^+^ and NAMN levels but not NMN; Pulse rate is strongly correlated with the increase in NAD^+^ level; No obvious adverse effects, and no significant changes in other indicators; Oral administration of NMN is safe	Japan	[140]
**/**	Double-blind, block-randomized, placebo-controlled study; 32 overweight or obese adults aged 55–80 y	Oral administration: 1000 mg once daily or twice daily for 14 d	NMN, NAD^+^, and NAD^+^ metabolome in blood and urine	1000 mg once or twice daily regimens were safe and associated with substantial dose-related increases in blood NAD levels and its metabolome	America	[122]
NCT03151239	Double-blind, randomized, placebo-controlled study; 25 postmenopausal and prediabetic women aged 55–75 y	Oral administration: 250 mg daily for 10 wk	NMN metabolites and NAD^+^ in plasma, PBMCs, and skeletal muscle; body composition and basal metabolic variables; skeletal muscle insulin sensitivity and signaling; skeletal muscle global transcriptome profile	↑ NAD^+^ and NMN metabolites in plasma; ↑ NMN metabolites in skeletal muscle but not NMN; ↑muscle insulin sensitivity, insulin signaling	America	[36]
ChiCTR2000035138	Double-blind, randomized, placebo-controlled study; 48 healthy recreationally trained runners aged 27–50 y	Oral administration: 300, 600 or 1200 mg daily for 6 wk	Body composition and cardiopulmonary function	↑aerobic capacity, enhanced O_2_ utilization of skeletal muscle; ↑VT in a dose-dependent manner; No obvious adverse symptoms and abnormal ECG	China	[145]
UMIN000036321	Double-blind, randomized, placebo-controlled study; 42 healthy old men aged ≥65 y	Oral administration: 250 mg daily for 12 wk	Clinical characteristics, blood and urine biochemical parameters, body composition, skeletal muscle mass, segmental lean	↑NAD^+^ and NAD^+^ metabolite levels in blood, improved muscle strength and performance, and no obvious adverse effects were observed	Japan	[141]
UMIN000038097	Double-blind, randomized, placebo-controlled study; 108 overweight or obese adults aged ≥65 y	Oral administration: 250 mg daily for 12 wk	body composition, muscle mass, bone mass, sleep quality, fatigue, physical performances	NMN intake in the afternoon is more effective in improving lower limb function and reducing drowsiness in older adults	Japan	[118]
NCT04228640	Nonblinded, nonrandomized, non–placebo-controlled study; 8 healthy men aged 45–60 y	Oral administration: 300 mg daily for 30–90 d	The telomere length of the PBMC	↑ telomere length of PBMC, which may be the potential molecular mechanisms of NMN for extending lifespan	China	[147]
NCT04228640	Double-blind, block-randomized, placebo-controlled study; 66 healthy participants aged 40–65 y	Oral administration: 300 mg NMN/d for 60 d	Blood cellular NAD^+^/NADH concentration in serum, six minutes walking endurance test, blood pressure, pulse pressure, SF-36 questionnaire, adverse events; blood biochemical parameters, HOMA-IR	↑NAD^+^/NADH levels in the serum, SF-36 score, minute walking endurance, and HOMA-IR index; ↓blood pressure, pulse pressure, and blood glucose; All test data did not have any statistically significant changes. However, the increase in NAD^+^/NADH levels in serum and the improvement in overall health and walking endurance were clinically significant	China	[146]
UMIN000043084	Double-blind, randomized, placebo-controlled study; 31 healthy participants aged 20–65 y	Oral administration: 1250 mg NMN/d for 4 wk	Safety evaluation of NMN oral administration in healthy adult men and women	Did not cause changes exceeding physiological variations (including anthropometry, hematological, biochemical, urine, and body composition)	Japan	[178]

2Py, N-methyl-2-pyridine-5-carboxamide; 4Py, N-methyl-4-pyridone-5-carboxamide; ECG, electrocardiogram; HOMA-IR, Homeostatic Model Assessment for Insulin Resistance; NAD, nicotinamide adenine dinucleotide; NAMN, nicotinic acid mononucleotide; NMN, nicotinamide mononucleotide; PBMC, peripheral blood mononuclear cell; SF-36, 36-Item Short Form Survey; VT, ventilatory threshold.

There is speculation that the human body can obtain NMN from daily food sources to support physiological function and NAD^+^ biosynthesis [100]. The amount of NMN obtained from food is likely ≤2 mg/d [36,100,179]. However, NAD^+^ concentration is reduced with age in human blood. Yang et al. [180] reported that the whole blood NAD^+^ concentration decreased gradually with aging in healthy men. Clement et al. [65] quantified changes in the NAD^+^ metabolome in plasma collected from healthy human subjects; their data showed NAD^+^ was significantly and negatively correlated with age from 20 to 87 y (correlation coefficient = −0.93). Hence, it is essential to supply NMN from nondietary sources to sustain proper NAD^+^ status. In human clinical trials with NMN, oral doses ranged from 100 mg/d to 2000 mg/d, with 250 mg/d being the most common dosage employed [35,36,118,140,141]. As a dietary supplement for antiaging and longevity, the dose of NMN available ranges from 50 to 500 mg per capsule in commercial products (data from Amazon.com and Ebay.com). Some consumers take 2 150-mg capsules daily [36]. The dose of NR available ranges from 100 to 2000 mg per softgel in commercial products (data from Amazon.com and Ebay.com). According to the dietary standards from the European Food Safety Authority, the tolerable upper intake level (UL) for NA is 10 mg/d, and the UL for NAM is 900 mg/d in adults [181]. The UL for niacin (including NA, NAM, and derivatives that exhibit the biological activity of NAM) is 35 mg/d (US National Academies of Science) [179]. Although there is currently no established UL for NR and NMN intake, based on molar equivalency, 900 mg of NAM equals 7.37 mmol, while 7.37 mmol of NR or 7.37 mmol of NMN equals 1889 mg and 2463 mg, respectively. Nevertheless, the safety of these higher doses cannot be determined without further toxicological studies and clinical trials involving larger cohorts. Establishing recommended safe levels for long-term administration is crucial. Still, compared with the RDA of vitamin B_3_, the supplemental dose of NMN is very high. Pellagra prevention is a different endpoint compared to optimizing NAD^+^ status to extend health span and reduce age-related dysfunction and disease. RDA is a minimum intake standard established to prevent a deficiency disease, so a direct comparison between them may not make much sense. However, NMN should be supplemented within a safe dose, at least below the UL.

A recent trend entails combining NMN with other antiaging agents. Compared to NMN alone, NMN combined with resveratrol and ginsenoside Rh2 and Rg3 showed better performance in increasing NAD^+^ concentrations in the heart and skeletal muscle of mice [182]. NMN has even been considered as an adjuvant to combat the COVID-19 pandemic; a study entitled “Study to evaluate the effect of NMN as an adjuvant to the standard of care on fatigue associated with COVID-19 infection” is currently in progress, and the results are of interest (NCT05175768).

There are several synthesis methods to obtain high-purity NMN [[183], [184], [185]], including chemical synthesis, microbial fermentation, and enzymatic synthesis. Chemical synthesis utilizes substrates such as 1,3,5-tri-O-benzoyl-β-D-ribofuranose, 1,2,3,5-tetra-O-acetyl-D-ribose, nicotinamide, ethyl nicotinate, and adenosine monophosphate. NMN is synthesized through Vorbruggen glycosylation, ketalization, phosphorylation, and ammonolysis [[186], [187], [188], [189]]. The disadvantages of this method include high cost, producing uncontrollable chiral by-products, multiple-step reactions, low yield, low purity, and the use of a large number of organic solvents, causing serious environmental damage [185,190]. On the other hand, biosynthesis is a relatively green and environment-friendly preparation method because it does not contain any organic solvent residue. Recently, several approaches have attempted to produce NMN in *Escherichia coli* but with low productivity and lack of practicality due to unintelligent mass transfer [183,191,192]. As for bioenzyme-catalyzed synthesis, it generally uses nicotinamide and phosphoribosyl pyrophosphate as substrates to generate NMN under the catalysis of nicotinamide phosphoribosyltransferase [190]. The phosphate group in NMN is primarily derived from energy-rich molecules like ATP or PRPP. However, the expensive cost of these precursors in the market results in a higher production cost for NMN [190]. Li et al. [185] designed an in vitro synthetic enzymatic biosystem to improve productivity further and reduce the production cost in one pot to produce NMN from low-cost starch and NAM. In a recent clinical trial, MetroBiotech, a Boston-based company, disclosed that its drug MIB-626 is a microcrystalline unique polymorph β-NMN formulation [122].

Although NMN has demonstrated antiaging properties in both cellular and animal models, there is a pressing need for additional clinical trials to determine its safety and efficacy in humans. Given its status as a top-selling antiaging product in recent years, human clinical trials on NMN have generated considerable interest. To date, 10 human clinical trials have been published (Table 2), with an additional 13 completed but unpublished trials and 11 ongoing trials (see Supplementary Table 2).

## Human Clinical Trials with NMN Supplementation

### Safety assessment

For the past few years, researchers have started to assess the safety and effects of NMN supplementation in humans to determine whether the effects observed in cells and animal models can be translated to humans. Thus far, we have identified 10 published human clinical trials, although more studies have yet to be published. The first clinical trial that assessed the safety of NMN in humans came from the Keio University School of Medicine (UMIN000021309) in 2016 [35]. To investigate the safety of NMN, a short-term study was conducted on 10 healthy men. During each visit, after overnight fasting, the participants orally consumed NMN capsules containing 100, 250, or 500 mg of NMN at 09:00. They were then monitored for 5 h at rest and were only allowed to drink water freely. The results of the study showed that the concentration of NMN metabolites (N-methyl-2-pyridine-5-carboxamide [2Py] and N-methyl-4-pyridone-5-carboxamide [4Py]) in human plasma increased as a result of NMN consumption, but there were no significant clinical symptoms, harmful effects, or changes in heart rate, blood pressure, oxygen saturation, or body temperature. The single oral administration of NMN up to 500 mg was safe and well-tolerated by the participants. Seven other studies conducted human clinical trials with the same NMN oral doses (250 mg once daily for 6 or 12 wk) or different doses (300, 600, and 1200 mg once daily for 6 wk; 300 mg once daily for 60 d) [36,118,140,141,[145], [146], [147]]. The highest NMN oral dose administered was 1000 mg twice daily for 14 d by Harvard Medical School [122]. The findings of these studies suggest that the administration of NMN orally is safe and has good tolerance. A recent investigation examined the safety of NMN in oral form (1250 mg/d for 4 wk) in 31 healthy individuals aged 20 to 65 and conducted an Ames test. The results revealed that NMN is a nonmutagenic substance that is safe and well-tolerated [178]. However, most selected participants were older individuals (age ≥55 y), and only 2 trials recruited middle-aged people (age <40 y) as partial participants, one from Guangzhou Sport University in China [145] and the other from the University of Toyama in Japan [140]. Nonetheless, it is believed that antiaging interventions should be initiated at a comparatively younger and healthier age than at a very old age, which will last longer. Therefore, there is a need for further investigation and determination of the safety and dietary reference intake of NMN in different age groups for long-term oral administration. Additionally, the number of participants in the human clinical trials conducted so far is limited. Eight studies included a range of 8 to 66 participants, which is primarily at the phase I clinical trial level. The University of Tsukuba in Japan conducted the most extensive trial with 108 older individuals as participants, utilizing a rigorous double-blind, randomized, placebo-controlled study method, which barely reached the level of a phase II clinical trial. In addition, 2 of these clinical trials evaluated the safety of oral NMN and the change of NAD^+^ and its metabolite concentrations in blood. Okabe et al. [140] reported that the concentrations of NAD^+^ and NAMN were significantly increased in whole blood after NMN intervention, but the levels of NMN, NAAD, NR, nicotinic acid nucleoside (NAR), NAM, NA, and *N*-methyl nicotinamide (MNAM) remained unchanged; however, the pulse rate exhibited a strong positive correlation with the increase of NAD^+^ concentration in blood. Although the exact reason is unclear, the association of pulse rate with energy consumption might be a direction for further investigation [140]. Pencina et al. [122] found that a higher dose of NMN was associated with a more pronounced increase in NMN and NAD^+^ concentration in the blood and the concentration of NAD^+^ metabolites (NAM and 2Py) in the urine. Three of the clinical trials included in the studies mentioned above evaluated both the safety of oral NMN and its potential antiaging effects, as well as the changes of NAD^+^ or its metabolite concentrations in blood, PBMCs, plasma, skeletal muscle, or urine.

Whether NMN is approved for marketing as a drug or dietary supplement, its safety for human consumption is always the most important issue. Although no clear side effects have been reported in existing human clinical trials, few studies have reported the possible toxic effects of NMN. For instance, Di Stefano et al. [193,194] reported a prodegenerative effect on axons after NMN supplementation in a chemotherapy-induced peripheral neuropathy mouse model. In addition, they revealed NMN-synthesizing enzyme as an important new therapeutic target in axonopathies. Nacarelli et al. [195] also found that NMN supplementation could enhance the proinflammatory SASP in oncogene-induced senescence cells and promote pancreatic ductal adenocarcinoma progression in a mouse model driven by oncogenic Kras. Moreover, a cell-permeant mimetic of NMN activated SARM1 to produce cyclic ADP-ribose and induced nonapoptotic cell death [196]. However, due to NMN’s limited toxicological data, future studies should focus more on this direction.

### Antiaging effect assessment

#### Sleep

Sleep quality is an important indicator of the health effects of any supplement. The first NMN human clinical trial assessed the sleep quality in 10 healthy Japanese men (age 40–60 y) via the Pittsburgh sleep quality index. It detected the levels of NMN and NAD^+^ metabolites (2Py and 4Py) in plasma. During the study, the researchers administered NMN (250 mg) or a placebo once a day for 6 or 12 wk to 20 healthy old men (age ≥65 y) and monitored their physiological muscle motility and blood NAD^+^ concentrations. The results showed a significant dose-dependent increase in NAD^+^ metabolites, but unfortunately, NMN was not detectable in the plasma samples. Moreover, there were no significant changes in the sleep quality score before and after NMN administration [35]. Owing to the poor sleep quality of older people, fatigue often occurs among them and more frequently in the afternoon. Therefore, sleep quality and fatigue in a lot of overweight or obese older people (age ≥ 65 y) were assessed via the Pittsburgh sleep quality index, self-reported sleep diary, and questionnaire [118]. The participants were divided into 2 groups: one took 250 mg NMN or placebo daily for 12 wk in the morning (after waking up until 12:00) and the other took 250 mg NMN or placebo daily for 12 wk in the afternoon (from 18:00 until bedtime). The results revealed no significant differences in sleep quality and fatigue tests before and after the intervention. However, the effect sizes of sleep quality and fatigue in the afternoon intervention group were larger than in the morning intervention group. Similar results were also found in the placebo-controlled group, in which the effect sizes in the afternoon placebo group were better than those in the morning placebo group [118]. The time-of-the-day-dependent manner of NMN intervention may be related to the circadian clock of NAD^+^ biosynthesis. Nakahata et al. [197] and Ramsey et al. [198] reported that NAD^+^ concentration (in serum-entrained mouse embryo fibroblasts and mouse liver) was the lowest between 12:00 to 20:00 every day, and CLOCK-SIRT1 and NAMPT regulated the circadian clock of NAD^+^ biosynthesis in mice.

#### Physical activity

Endurance exercise increases aerobic capacity by improving mitochondrial function, vascular endothelium function, and capillary muscle density [199]. In addition, NMN has also been reported to improve mitochondrial function in various metabolic organs (such as skeletal muscle), vascular endothelium function, neoangiogenesis, capillary density, blood flow, and soluble oxygen levels in rodents [22,23,148,174]. Igarashi et al. [141] investigated the effects of NMN intervention on the physical activity of older individuals. They observed the physiological muscle motility and blood NAD^+^ concentrations in 20 healthy older men (age ≥65 y), and NMN (250 mg) or placebo was administered once a day for 6 or 12 wk [141]. The NMN intervention did not have an impact on insulin sensitivity, skeletal muscle, and visceral fat mass. However, it was found to significantly improve gait speed, left grip strength, and the frequency of the 30-s chair-stand test [141]. Oral NMN supplementation effectively increased NMN and NAD^+^ concentrations in blood, and an increase in NR level was also observed, indicating that NMN might be converted into NR by CD73 [141]. It should be noted that oral NMN also significantly increased the level of NAMN and NAR, which was not a route for converting NMN to NAD^+^ [141]. The increase of NAD^+^ concentration in blood may lead to the deamidation of NMN and, ultimately, the formation of NAM; besides, the deamidation of NMN by intestinal microflora may be another mechanism [141]. In summary, Igarashi’s research demonstrated that NMN was an efficient NAD^+^ booster for preventing aging-related muscle dysfunctions in humans [141]. Kim et al. [118] evaluated the effect of oral NMN on physical performance in older individuals, including grip strength, 5-times sit-to-stand (5-STS), timed up and go, and a 5-m habitual walk. The 5-STS test results for all groups postintervention showed significant improvement compared to those before the intervention. The effect size of the NMN intervention in the afternoon (d = 0.72) was larger than that of the morning intervention (d = 0.40). No significant improvement was observed in other test items. Overall, these findings suggest the potential of NMN to improve physical performance in older adults.

The effects of NMN intervention on the physical activity of middle-aged people were investigated by the Department of Sports Medicine, Guangzhou Sport University, China [145] and Effepharm (Shanghai) Co., Ltd, China [146]. Liao et al. [145] conducted a double-blind, randomized, placebo-controlled human clinical trial, which included 48 young and middle-aged recreationally trained runners (aged 27–50 y). The participants underwent randomization into 4 groups, with each group receiving either 300 mg, 600 mg, 1200 mg NMN or a placebo orally for 6 wk. Concurrently, all participants performed aerobic exercise, including running and cycling, with 40- to 60-min training sessions 5 to 6 times each week while taking the NMN orally [145]. In addition, cardiopulmonary exercise testing was performed to assess the aerobic capacity of the runners; NMN intervention significantly increased oxygen consumption in ventilatory threshold 1 (VT1) and improved energy consumption in VT1 and VT2. These results indicate that physical training combined with oral NMN administration could be a new strategy to improve athletes’ performance [145]. Huang et al. [146] conducted a 6-min walking endurance test for older people (age 40–65 y) after NMN intervention (300 mg/d for 60 d). On day 30 of the treatment, the NMN intervention and placebo groups exhibited a 4.3% and 3.9% increase in walking endurance, respectively. When the same treatment was continued for up to 60 d, the NMN intervention group showed a further rise of 6.5%, whereas the placebo group showed no additional increase, remaining at 3.9%. Although the difference between the NMN and placebo intervention groups was not statistically significant in the 6-min walking endurance test, it was evident that the NMN intervention group showed sustained improvement in walking endurance from 30 to 60 d of treatment [146].

#### Nervous system-related

Irie et al. [35] assessed ophthalmic parameters, including visual acuity, functional visual acuity, intraocular pressure measurement, and meniscometry. The results showed no significant changes in ophthalmic parameters before and after NMN administration. In addition, Igarashi et al. [141] conducted a hearing test and a relatively simple cognitive function test. Furthermore, the right auditory ability of older men significantly improved, and the authors hypothesized that the underlying mechanism could be similar to that observed in mice, where NMN supplementation activates the SIRT3 protein and regulates the reduced/oxidized glutathione ratio in the mitochondria [141,200,201]. However, no effect was observed in overall cognitive function in the mini-mental state examination Japanese and the Japanese version of the Montreal cognitive assessment [141].

#### Diabetes

Diabetes is a leading cause of blindness, amputations, heart disease, kidney failure, and premature death. So far, there is no effective method to cure diabetes. A study led by Yoshino et al. [36] showed that NMN could increase muscle insulin sensitivity, insulin signaling, and remodeling in women with prediabetes who are overweight or obese. A total of 25 postmenopausal women with prediabetes were randomly assigned to either the placebo group (*n* = 12, 250 mg/d) or the oral NMN group (*n* = 13, 250 mg/d) for 10 wk. The study assessed the following indicators: *1*) NMN metabolites and NAD^+^ concentrations in plasma, PBMCs and skeletal muscle; *2*) body composition and basal metabolic indexes; *3*) the impact of NMN on skeletal muscle insulin sensitivity and signaling; and *4*) the effects of NMN on the skeletal muscle global transcriptome profile [36]. Surprisingly, after NMN intervention, participants’ insulin sensitivity was improved by 25 ± 7% [36]. Interestingly, it was also found that a series of downstream signals of the insulin pathway, including the phosphorylation of AKT at Ser473 and Thr308, was triggered and the expression of platelet-derived growth factor (PDGF) receptor β and other muscle remodeling-associated genes were upregulated. Since the PDGF signaling pathway has been reported to enhance insulin-stimulated AKT phosphorylation and glucose transport in skeletal muscle and multiple cell types in previous studies [202,203], this provided a possible explanation for the effect of NMN in enhancing muscle insulin sensitivity. In addition, NAD^+^ concentrations did not change in skeletal muscle but increased significantly in PBMCs, and the NAD^+^ metabolites (2Py and 4Py) significantly increased both in PBMCs and skeletal muscle [36]. A sex difference was observed in the effect of NMN on glucose tolerance in diabetic mice and female mice were more sensitive than male mice [24]. Although the cause of sex difference is still unclear, the results showed that NMN treatment could improve impaired glucose tolerance by improving insulin sensitivity or insulin secretion [24]. Moreover, the effect of sex differences on glucose tolerance also needs to be further verified in human clinical trials.

#### Telomere

Telomere shortening is an important biomarker for aging [204]. NMN has been reported to maintain telomere length in the liver of mouse [205]. In another study, Niu et al. [147] examined the changes in telomere length in 8 middle-aged men (aged 45–60 y) before and after oral administration of 300 mg/d NMN. They discovered that NMN supplementation resulted in a nearly doubled telomere length in PBMCs within 90 d, indicating a potential antiaging effect. The underlying mechanism of NMN on elongating telomere length may be associated with the increased NAD^+^ concentration in liver [24,205], stabilizing telomere and preventing tissue damage and fibrosis in a partially sirtuin-1-dependent manner [205].

### Completed but Unpublished and Ongoing Clinical Trials

We also identified completed but unpublished clinical trials and ongoing clinical trials from clinicaltrials.gov (https://www.clinicaltrials.gov/), WHO International Clinical Trials Registry Platform (https://trialsearch.who.int), and UMIN-CTR Search Clinical Trials (https://center6.umin.ac.jp/cgi-open-bin/ctr_e/index.cgi?function=02). Furthermore, 13 completed but unpublished clinical trials and 11 ongoing clinical trials were identified (Supplementary Table 2). The objectives of these trials encompassed the following aspects: *1*) assessing the safety and metabolic kinetics of NMN nutrition intervention in healthy adults (18–70 y); *2*) investigating the effects of NMN nutrition intervention on various diseases (including diabetes, chronic disease, hypertension, polycystic ovary syndrome, and premature ovarian failure); *3*) observing the antiaging effects of NMN nutrition intervention on the skin (including fine lines and wrinkles, eye bags, dark circles, skin texture, moisture, puffiness, and brightness); *4*) studying changes in various hormonal levels and aging markers, male fertility indicators, cardiovascular and metabolic functions, and physical activity; *5*) comparing the impact of NMN and other NAD^+^ precursors on blood NAD^+^ metabolome. Although each clinical trial must be registered in a publicly accessible database, there is no obligation to publish the results. This is unfortunate, as results from completed but unpublished clinical trials would provide useful information for scrutinizing NMN nutrition intervention’s safety and antiaging effects. Thus, it is strongly recommended that the results of any reasonable clinical trial should be published, and this should be the responsibility of the researchers and the funding sources, including the food and pharmaceutical industries.

## Conclusions and Challenges

Existing human clinical trials suggest that oral NMN administration is generally safe, and although only a limited number of indicators were studied, the results suggest that NMN has potential as an antiaging agent. However, there are still obstacles that need to be addressed before NMN-containing products can be confidently marketed.

1. Longer, larger and better-designed human trials are needed to investigate NMN administration’s safe dosage, tolerance and frequency.

Humans usually take supplements for a long time and sometimes for most of their lifespan. Thus, the long-term safety issue should be addressed about NMN supplementation. Also, a larger/more diverse population should be examined, as certain adverse effects could only be observed in a very small number of people. Furthermore, it remains to be seen whether the beneficial effects of NMN were only limited to a specific group or the general population. For instance, NMN supplementation increased skeletal muscle insulin signaling, insulin sensitivity, and muscle remodeling in postmenopausal women with prediabetes, how about other populations? Finally, better-controlled clinical trials will avoid biased results.

2. More comprehensive studies are needed to elucidate the beneficial effects of NMN and underlying mechanisms fully. In addition, mechanistic toxicological studies are also warranted.

In the existing human clinical trials, very limited indicators were examined; in particular, nutritionally relevant endpoints were absent. For example, whether NMN supplementation could improve the absorption of nutrients, whether and how NMN affects the activity of the various metabolic enzymes; and as a hot research area in recent years, whether NMN has any influence on the gut microbiota and whether gut microbiota mediates the function of NMN. Such topics are all worthy of further investigation. Thus, omics techniques (such as the evaluation of transcriptome, proteome, and metabolome) should be used to establish the toxicology, beneficial effects and safety dose ranges of NMN in humans, including different age groups, health status and sex. Also, even though NAD^+^ and NAD^+^ metabolites can be detected in the blood after oral NMN administration, the biological effects may differ in different tissues and organs. Thus, the biological effects on different tissues and organs should be considered in future studies. More importantly, a better understanding of the molecular mechanism of the action of NMN is a prerequisite for its human applications, which will help to avoid unwanted side effects.

3. Some fundamental issues regarding NAD^+^ and NMN must be carefully addressed. Different ethnic groups, age groups, gender groups and dietary pattern groups may have different ‘normal’ NAD^+^ concentrations. Without a clear definition of the ‘normal’ concentration, many results from different studies may not be applied to other populations. Thus, a large-scale baseline measurement of NAD^+^ and NADome in multiple age groups and regions is necessary to establish the ‘golden standard.’ Furthermore, many questions remained to be answered, e.g., whether and to what extent blood and tissue NAD^+^ concentration should be considered as biomarker of status; the need for population-based studies to determine whether blood and tissue concentrations can be considered surrogates for dysfunction or risk for chronic disease; and how NMN (or other precursors) supplementation may impact these. Also, large epidemiological studies need to show a clear relationship between NAD^+^ ‘deficiency’ and age-associated health outcomes. As noted before, there were currently few such studies, and without a clear association, the potential of NMN, or other NAD^+^ precursors, is greatly undermined.

Finally, an important issue to consider is the excessive hype surrounding NMN in the market. For example, some have touted NMN as a solution to skin aging, and while a recent study showed promising results in mice, further research is needed before conclusions can be drawn about its effectiveness in humans [33]. Another study showed that NMN reduced melanogenesis in aged melanocytes by downregulating the signaling of melanogenesis-associated receptors [206]. Nevertheless, there have yet to be any results obtained from those human clinical trials examining the skin antiaging effect by either oral or external NMN intervention. Furthermore, it is well known that NMN cannot pass through the skin barrier directly due to its high water solubility. However, many NMN skin care products, including essence, facial mask, moisturizing water, and sunscreen, are widely sold. Thus, there is an urgent need to conduct appropriate clinical trials to determine the effects and safety of NMN supplements in different aspects so that their potential benefits can be realized in more people.

## References

[1] (2019). WHO launches digital app to improve care for older people [Internet].

[2] (2022). GBD 2019 Ageing Collaborators, Global, regional, and national burden of diseases and injuries for adults 70 years and older: systematic analysis for the global burden of disease 2019 study. BMJ.

[3] Cai Y.S., Song W., Li J.M., Jing Y., Liang C.Q., Zhang L.Y. (2022). The landscape of aging. Sci. China Life Sci..

[4] Schmauck-Medina T., Molière A., Lautrup S., Zhang J., Chlopicki S., Madsen H.B. (2022). New hallmarks of ageing: a 2022 Copenhagen ageing meeting summary. Aging (Albany NY).

[5] Cai A.P., Zhou D., Liu L., Zhou Y.L., Tang S.T., Feng Y.Q. (2021). Age-related alterations in cardiac and arterial structure and function in hypertensive women and men. J. Clin. Hypertens. (Greenwich).

[6] Xiang Q.Y., Tian F., Xu J., Du X., Zhang S.L., Liu L. (2022). New insight into dyslipidemia-induced cellular senescence in atherosclerosis. Biol. Rev. Camb. Philos. Soc..

[7] Chrienova Z., Nepovimova E., Kuca K. (2021). The role of mTOR in age-related diseases. J. Enzyme Inhib. Med. Chem..

[8] Čater M., Križančić Bombek L. (2022). Protective role of mitochondrial uncoupling proteins against age-related oxidative stress in type 2 diabetes mellitus. Antioxidants (Basel).

[9] Wang Y.Q., Xue M.Z., Xia F.Q., Zhu L.Q., Jia D.K., Gao Y. (2022). Long non-coding RNA GAS5 in age-related diseases. Curr. Med. Chem..

[10] Segen V., Ying J., Morgan E., Brandon M., Wolbers T. (2022). Path integration in normal aging and Alzheimer’s disease. Trends Cogn. Sci..

[11] GeneHarbor Herbalmax, Formulas Genex (2021). https://www.precisionreports.co/global-nicotinamide-mononucleotide-nmn-sales-market-17556260.

[12] Verdin E. (2015). NAD^+^ in aging, metabolism, and neurodegeneration. Science.

[13] Kane A.E., Sinclair D.A. (2018). Sirtuins and NAD^+^ in the development and treatment of metabolic and cardiovascular diseases. Circ. Res..

[14] Camacho-Pereira J., Tarragó M.G., Chini C.C.S., Nin V., Escande C., Warner G.M. (2016). CD38 dictates age-related NAD decline and mitochondrial dysfunction through an SIRT3-dependent mechanism. Cell Metab.

[15] Yang Y., Sauve A.A. (2016). NAD^+^ metabolism: bioenergetics, signaling and manipulation for therapy. Biochim. Biophys. Acta.

[16] Cantó C., Menzies K.J., Auwerx J. (2015). NAD^+^ metabolism and the control of energy homeostasis: a balancing act between mitochondria and the nucleus. Cell Metab.

[17] Zheng T., Xu S.Y., Zhou S.Q., Lai L.Y., Li L. (2013). Nicotinamide adenine dinucleotide (NAD^+^) repletion attenuates bupivacaine-induced neurotoxicity. Neurochem. Res..

[18] Preyat N., Rossi M., Kers J., Chen L., Bertin J., Gough P.J. (2016). Intracellular nicotinamide adenine dinucleotide promotes TNF-induced necroptosis in a sirtuin-dependent manner. Cell Death Differ.

[19] Wilk A., Hayat F., Cunningham R., Li J.F., Garavaglia S., Zamani L. (2020). Extracellular NAD^+^ enhances PARP-dependent DNA repair capacity independently of CD73 activity. Sci. Rep..

[20] Sánchez-Ramírez E., Ung T.P.L., Alarcón Del Carmen A., del Toro-Ríos X., Fajardo-Orduña G.R., Noriega L.G. (2022). Coordinated metabolic transitions and gene expression by NAD^+^ during adipogenesis. J. Cell Biol..

[21] Elvehjem C.A., Madden R.J., Strong F.M., Woolley D.W. (1937). Relation of nicotinic acid and nicotinic acid amide to canine black tongue. J. Am. Chem. Soc..

[22] Covarrubias A.J., Perrone R., Grozio A., Verdin E. (2021). NAD^+^ metabolism and its roles in cellular processes during ageing. Nat. Rev. Mol. Cell Biol..

[23] Houtkooper R.H., Auwerx J. (2012). Exploring the therapeutic space around NAD^+^. J. Cell Biol..

[24] Yoshino J., Mills K.F., Yoon M.J., Imai S.I. (2011). Nicotinamide mononucleotide, a key NAD^+^ intermediate, treats the pathophysiology of diet- and age-induced diabetes in mice. Cell Metab.

[25] Poljsak B., Kovač V., Milisav I. (2020). Healthy lifestyle recommendations: do the beneficial effects originate from NAD^+^ amount at the cellular level?. Oxid. Med. Cell. Longev..

[26] Menssen A., Hydbring P., Kapelle K., Vervoorts J., Diebold J., Lüscher B. (2012). The c-MYC oncoprotein, the NAMPT enzyme, the SIRT1-inhibitor DBC1, and the SIRT1 deacetylase form a positive feedback loop. Proc. Natl. Acad. Sci. U. S. A..

[27] Massudi H., Grant R., Guillemin G.J., Braidy N. (2012). NAD^+^ metabolism and oxidative stress: the golden nucleotide on a crown of thorns. Redox Rep.

[28] Lin S.J., Ford E., Haigis M., Liszt G., Guarente L. (2004). Calorie restriction extends yeast life span by lowering the level of NADH. Genes Dev.

[29] Reiten O.K., Wilvang M.A., Mitchell S.J., Hu Z.P., Fang E.F. (2021). Preclinical and clinical evidence of NAD^+^ precursors in health, disease, and ageing. Mech. Ageing Dev..

[30] Rajman L., Chwalek K., Sinclair D.A. (2018). Therapeutic potential of NAD-boosting molecules: the in vivo evidence. Cell Metab.

[31] Gomes A.P., Price N.L., Ling A.J.Y., Moslehi J.J., Montgomery M.K., Rajman L. (2013). Declining NAD^+^ induces a pseudohypoxic state disrupting nuclear-mitochondrial communication during aging. Cell.

[32] Das A., Huang G.X., Bonkowski M.S., Longchamp A., Li C., Schultz M.B. (2019). Impairment of an endothelial NAD^+^-H_2_S signaling network is a reversible cause of vascular aging. Cell.

[33] Fang T., Yang J., Liu L., Xiao H., Wei X. (2021). Nicotinamide mononucleotide ameliorates senescence in alveolar epithelial cells. MedComm.

[34] Ru M., Wang W.W., Zhai Z.Y., Wang R.X., Li Y.M., Liang J. (2022). Nicotinamide mononucleotide supplementation protects the intestinal function in aging mice and d-galactose induced senescent cells. Food Funct.

[35] Irie J., Inagaki E., Fujita M., Nakaya H., Mitsuishi M., Yamaguchi S. (2020). Effect of oral administration of nicotinamide mononucleotide on clinical parameters and nicotinamide metabolite levels in healthy Japanese men. Endocr. J..

[36] Yoshino M., Yoshino J., Kayser B.D., Patti G.J., Franczyk M.P., Mills K.F. (2021). Nicotinamide mononucleotide increases muscle insulin sensitivity in prediabetic women. Science.

[37] Revollo J.R., Körner A., Mills K.F., Satoh A., Wang T., Garten A. (2007). Nampt/PBEF/Visfatin regulates insulin secretion in beta cells as a systemic NAD biosynthetic enzyme. Cell Metab.

[38] Lin H. (2007). Nicotinamide adenine dinucleotide: beyond a redox coenzyme. Org. Biomol. Chem..

[39] Cuenoud B., Ipek Ö., Shevlyakova M., Beaumont M., Cunnane S.C., Gruetter R. (2020). Brain NAD is associated with ATP energy production and membrane phospholipid turnover in humans. Front. Aging Neurosci..

[40] Walker M.A., Tian R. (2018). NAD(H) in mitochondrial energy transduction: implications for health and disease. Curr. Opin. Physiol..

[41] Dudev T., Lim C. (2010). Factors controlling the mechanism of NAD^+^ non-redox reactions. J. Am. Chem. Soc..

[42] Scheibye-Knudsen M., Mitchell S.J., Fang E.F., Iyama T., Ward T., Wang J. (2014). A high-fat diet and NAD^+^ activate Sirt1 to rescue premature aging in Cockayne syndrome. Cell Metab.

[43] Yu P.L., Liu Z.M., Yu X.F., Ye P.W., Liu H., Xue X.W. (2019). Direct gating of the TRPM2 channel by cADPR via specific interactions with the ADPR binding pocket. Cell Rep.

[44] Zhang P., Kishimoto Y., Grammatikakis I., Gottimukkala K., Cutler R.G., Zhang S. (2019). Senolytic therapy alleviates abeta-associated oligodendrocyte progenitor cell senescence and cognitive deficits in an Alzheimer’s disease model. Nat. Neurosci..

[45] Kauppinen T.M., Suh S.W., Higashi Y., Berman A.E., Escartin C., Won S.J. (2011). Poly(ADP-ribose)polymerase-1 modulates microglial responses to amyloid β. J. Neuroinflammation.

[46] Ernst I.M., Fliegert R., Guse A.H. (2013). Adenine dinucleotide second messengers and T-lymphocyte calcium signaling. Front. Immunol..

[47] Turunc Bayrakdar E., Uyanikgil Y., Kanit L., Koylu E., Yalcin A. (2014). Nicotinamide treatment reduces the levels of oxidative stress, apoptosis, and PARP-1 activity in Aβ(1-42)-induced rat model of Alzheimer's disease. Free Radic. Res..

[48] Elkhal A., Biefer H.R.C., Heinbokel T., Uehara H., Quante M., Seyda M. (2016). NAD^+^ plus regulates Treg cell fate and promotes allograft survival via a systemic IL-10 production that is CD4^+^ CD25^+^ Foxp3^+^ T cells independent. Sci. Rep..

[49] Wu X.L., Wang P., Liu Y.H., Xue Y.X. (2014). Effects of poly (ADP-ribose) polymerase inhibitor 3-aminobenzamide on blood-brain barrier and dopaminergic neurons of rats with lipopolysaccharide-induced Parkinson’s disease. J. Mol. Neurosci..

[50] Mandir A.S., Przedborski S., Jackson-Lewis V., Wang Z.Q., Simbulan-Rosenthal C.M., Smulson M.E. (1999). Poly(ADP-ribose) polymerase activation mediates 1-methyl-4-phenyl-1, 2,3,6-tetrahydropyridine (MPTP)-induced parkinsonism. Proc. Natl. Acad. Sci. U S A.

[51] Kim T.W., Cho H.M., Choi S.Y., Suguira Y., Hayasaka T., Setou M. (2013). (ADP-ribose) polymerase 1 and AMP-activated protein kinase mediate progressive dopaminergic neuronal degeneration in a mouse model of Parkinson’s disease. Cell Death Dis.

[52] Hou Y., Lautrup S., Cordonnier S., Wang Y., Croteau D.L., Zavala E. (2018). NAD^+^ supplementation normalizes key Alzheimer’s features and DNA damage responses in a new AD mouse model with introduced DNA repair deficiency. Proc. Natl. Acad. Sci. U S A.

[53] Yao Z.W., Yang W.H., Gao Z.G., Jia P. (2017). Nicotinamide mononucleotide inhibits JNK activation to reverse Alzheimer disease. Neurosci. Lett..

[54] Liszt G., Ford E., Kurtev M., Guarente L. (2005). Mouse Sir2 homolog SIRT6 is a nuclear ADP-ribosyltransferase. J. Biol. Chem..

[55] Wang X., Hu X., Yang Y., Takata T., Sakurai T. (2016). Nicotinamide mononucleotide protects against beta-amyloid oligomer-induced cognitive impairment and neuronal death. Brain Res.

[56] Chi Y.L., Sauve A.A. (2013). Nicotinamide riboside, a trace nutrient in foods, is a Vitamin B3 with effects on energy metabolism and neuroprotection. Curr. Opin. Clin. Nutr. and Metab. Care.

[57] Torti M., Bertoni A., Canobbio I., Sinigaglia F., Balduini C. (1999). Hydrolysis of NADP^+^ by platelet CD38 in the absence of synthesis and degradation of cyclic ADP-ribose 2’-phosphate. FEBS Lett.

[58] Sorrentino V., Omani M.R., Ouchiroud L.M., Beck J.S., Zhang H.B., D’Amico D. (2017). Enhancing mitochondrial proteostasis reduces amyloid-β proteotoxicity. Nature.

[59] Fang E.F., Hou Y.J., Palikaras K., Adriaanse B.A., Kerr J.S., Yang B.M. (2019). Mitophagy inhibits amyloid-beta and tau pathology and reverses cognitive deficits in models of Alzheimer’s disease. Nat. Neurosci..

[60] Lehmann S., Loh S.H.Y., Martins L.M. (2017). Enhancing NAD^+^ salvage metabolism is neuroprotective in a PINK1 model of Parkinson’s disease. Biol. Open.

[61] Fagnoni F.F., Vescovini R., Mazzola M., Bologna G., Nigro E., Lavagetto G. (1996). Expansion of cytotoxic CD8^+^ CD28^−^ T cells in healthy ageing people, including centenarians. Immunology.

[62] Weng N.P., Akbar A.N., Goronzy J. (2009). CD28^−^ T cells: their role in the age-associated decline of immune function. Trends Immunol.

[63] Jia H.Q., Li X., Gao H.X., Feng Z., Li X., Zhao L. (2008). High doses of nicotinamide prevent oxidative mitochondrial dysfunction in a cellular model and improve motor deficit in a Drosophila model of Parkinson’s disease. J. Neurosci. Res..

[64] Minhas P.S., Liu L., Moon P.K., Joshi A.U., Dove C., Mhatre S. (2019). Macrophage de novo NAD^+^ synthesis specifies immune function in aging and inflammation. Nat. Immunol..

[65] Clement J., Wong M., Poljak A., Sachdev P., Braidy N. (2019). The plasma NAD^+^ metabolome is dysregulated in “normal” aging. Rejuvenation Res.

[66] Massudi H., Grant R., Braidy N., Guest J., Farnsworth B., Guillemin G.J. (2012). Age-associated changes in oxidative stress and NAD^+^ metabolism in human tissue. PLOS ONE.

[67] Zhou C.C., Yang X., Hua X., Liu J., Fan M.B., Li G.Q. (2016). Hepatic NAD^+^ deficiency as a therapeutic target for nonalcoholic fatty liver disease in ageing. Br. J. Pharmacol..

[68] Janssens G.E., Grevendonk L., Perez R.Z., Schomakers B.V., Bosch J.V., Geurts J.W. (2022). Healthy aging and muscle function are positively associated with NAD^+^ abundance in humans. Nat. Aging.

[69] Bagga P., Hariharan H., Wilson N.E., Beer J.C., Shinohara R.T., Elliott M.A. (2020). Single-voxel ^1^H MR spectroscopy of cerebral nicotinamide adenine dinucleotide (NAD^+^) in humans at 7T using a 32-channel volume coil. Magn. Reson. Med..

[70] Zhu X.H., Lu M., Lee B.Y., Ugurbil K., Chen W. (2015). In vivo NAD assay reveals the intracellular NAD contents and redox state in healthy human brain and their age dependences. Proc. Natl. Acad. Sci. U S A.

[71] Fang E.F., Lautrup S., Hou Y.J., Demarest T.G., Croteau D.L., Mattson M.P. (2017). NAD^+^ in aging: molecular mechanisms and translational implications. Trends Mol. Med..

[72] Imai S., Guarente L. (2014). NAD^+^ and sirtuins in aging and disease. Trends Cell Biol.

[73] Zha S.Y., Li Z., Cao Q., Wang F., Liu F. (2018). PARP1 inhibitor (PJ34) improves the function of aging-induced endothelial progenitor cells by preserving intracellular NAD^+^ levels and increasing SIRT1 activity. Stem Cell Res. Ther..

[74] Wang Q.H., Li Y., Dou D.Y., Wang R., Jiang T.T., Wang L. (2021). Nicotinamide mononucleotide-elicited NAMPT signaling activation aggravated adjuvant-induced arthritis in rats by affecting peripheral immune cells differentiation. Int. Immunopharmacol..

[75] Abdellatif M., Sedej S., Kroemer G. (2021). NAD^+^ metabolism in cardiac health, aging, and disease. Circulation.

[76] She J., Sheng R., Qin Z.H. (2021). Pharmacology and potential implications of nicotinamide adenine dinucleotide precursors. Aging Dis.

[77] Fan L., Cacicedo J.M., Ido Y.S. (2020). Impaired nicotinamide adenine dinucleotide (NAD^+^) metabolism in diabetes and diabetic tissues: implications for nicotinamide-related compound treatment. J. Diabetes Investig..

[78] Colombo G., Gelardi E.L.M., Balestrero F.C., Moro M., Travelli C., Genazzani A.A. (2021). Insight into nicotinamide adenine dinucleotide homeostasis as a targetable metabolic pathway in colorectal cancer. Front. Pharmacol..

[79] McReynolds M.R., Chellappa K., Baur J.A. (2020). Age-related NAD^+^ decline. Exp. Gerontol..

[80] Ma X.R., Zhu X.D., Xiao Y.J., Gu H.M., Zheng S.S., Li L. (2022). Restoring nuclear entry of Sirtuin 2 in oligodendrocyte progenitor cells promotes remyelination during ageing. Nat. Commun..

[81] Luo C., Ding W., Yang C., Zhang W., Liu X., Deng H. (2022). Nicotinamide mononucleotide administration restores redox homeostasis via the Sirt3-Nrf2 axis and protects aged mice from oxidative stress-induced liver injury. J. Proteome Res..

[82] Ramanathan C., Lackie T., Williams D.H., Simone P.S., Zhang Y.F., Bloomer R.J. (2022). Oral administration of nicotinamide mononucleotide increases nicotinamide adenine dinucleotide level in an animal brain. Nutrients.

[83] Zheng S.L., Wang D.S., Dong X., Guan Y.F., Qi Q., Hu W.J. (2023). Distribution of nicotinamide mononucleotide after intravenous injection in normal and ischemic stroke mice. Curr. Pharm. Biotechnol..

[84] Shen C.Y., Li X.Y., Ma P.Y., Li H.L., Xiao B., Cai W.F. (2022). Nicotinamide mononucleotide (NMN) and NMN-rich product supplementation alleviate p-chlorophenylalanine-induced sleep disorders. J. Funct. Foods.

[85] Ma D., Hu L., Wang J., Luo M., Liang A., Lei X. (2022). Nicotinamide mononucleotide improves spermatogenic function in streptozotocin-induced diabetic mice via modulating the glycolysis pathway. Acta Biochim. Biophys. Sin. (Shanghai).

[86] Zhou X., Du H.H., Long X., Pan Y., Hu J., Yu J. (2021). Beta-nicotinamide mononucleotide (NMN) administrated by intraperitoneal injection mediates protection against UVB-induced skin damage in mice. J. Inflamm. Res..

[87] Chen X.H., Amorim J.A., Moustafa G.A., Lee J.J., Yu Z., Ishihara K. (2020). Neuroprotective effects and mechanisms of action of nicotinamide mononucleotide (NMN) in a photoreceptor degenerative model of retinal detachment. Aging US.

[88] Miao Y., Cui Z., Gao Q., Rui R., Xiong B. (2020). Nicotinamide mononucleotide supplementation reverses the declining quality of maternally aged oocytes. Cell Rep.

[89] Kiss T., Giles C.B., Tarantini S., Yabluchanskiy A., Balasubramanian P., Gautam T. (2020). Nicotinamide mononucleotide (NMN) supplementation promotes anti-aging miRNA expression profile in the aorta of aged mice, predicting epigenetic rejuvenation and anti-atherogenic effects. Geroscience.

[90] Kiss T., Nyúl-Tóth Á., Balasubramanian P., Tarantini S., Ahire C., Yabluchanskiy A. (2020). Nicotinamide mononucleotide (NMN) supplementation promotes neurovascular rejuvenation in aged mice: transcriptional footprint of SIRT1 activation, mitochondrial protection, anti-inflammatory, and anti-apoptotic effects. Geroscience.

[91] Liang H., Gao J., Zhang C., Li C., Wang Q., Fan J. (2019). Nicotinamide mononucleotide alleviates aluminum induced bone loss by inhibiting the TXNIP-NLRP3 inflammasome. Toxicol. Appl. Pharmacol..

[92] Hosseini L., Farokhi-Sisakht F., Badalzadeh R., Khabbaz A., Mahmoudi J., Sadigh-Eteghad S. (2019). Nicotinamide mononucleotide and melatonin alleviate aging-induced cognitive impairment via modulation of mitochondrial function and apoptosis in the prefrontal cortex and hippocampus. Neuroscience.

[93] Tarantini S., Valcarcel-Ares M.N., Toth P., Yabluchanskiy A., Tucsek Z., Kiss T. (2019). Nicotinamide mononucleotide (NMN) supplementation rescues cerebromicrovascular endothelial function and neurovascular coupling responses and improves cognitive function in aged mice. Redox Biol.

[94] Sims C.A., Guan Y.X., Mukherjee S., Singh K., Botolin P., Davila A. (2018). Nicotinamide mononucleotide preserves mitochondrial function and increases survival in hemorrhagic shock. JCI Insight.

[95] Wei C.C., Kong Y.Y., Hua X., Li G.Q., Zheng S.L., Cheng M.H. (2017). NAD replenishment with nicotinamide mononucleotide protects blood-brain barrier integrity and attenuates delayed tissue plasminogen activator-induced haemorrhagic transformation after cerebral ischaemia. Br. J. Pharmacol..

[96] Wei C.C., Kong Y.Y., Li G.Q., Guan Y.F., Wang P., Miao C.Y. (2017). Nicotinamide mononucleotide attenuates brain injury after intracerebral hemorrhage by activating Nrf2/HO-1 signaling pathway. Sci. Rep..

[97] Martin A.S., Abraham D.M., Hershberger K.A., Bhatt D.P., Mao L., Cui H.X. (2017). Nicotinamide mononucleotide requires SIRT3 to improve cardiac function and bioenergetics in a Friedreich’s ataxia cardiomyopathy model. JCI Insight.

[98] Zhang R., Shen Y., Zhou L., Sangwung P., Fujioka H., Zhang L. (2017). Short-term administration of nicotinamide mononucleotide preserves cardiac mitochondrial homeostasis and prevents heart failure. J. Mol. Cell. Cardiol..

[99] De Picciotto N.E., Gano L.B., Johnson L.C., Martens C.R., Sindler A.L., Mills K.F. (2016). Nicotinamide mononucleotide supplementation reverses vascular dysfunction and oxidative stress with aging in mice. Aging Cell.

[100] Mills K.F., Yoshida S., Stein L.R., Grozio A., Kubota S., Sasaki Y. (2016). Long-term administration of nicotinamide mononucleotide mitigates age-associated physiological decline in mice. Cell Metab.

[101] Uddin G.M., Youngson N.A., Sinclair D.A., Morris M.J. (2016). Head to head comparison of short-term treatment with the NAD^+^ precursor nicotinamide mononucleotide (NMN) and 6 weeks of exercise in obese female mice. Front. Pharmacol..

[102] Lee C.F., Chavez J.D., Garcia-Menendez L., Choi Y., Roe N.D., Chiao Y.A. (2016). Normalization of NAD^+^ redox balance as a therapy for heart failure. Circulation.

[103] Park J.H., Long A., Owens K., Kristian T. (2016). Nicotinamide mononucleotide inhibits postischemic NAD^+^ degradation and dramatically ameliorates brain damage following global cerebral ischemia. Neurobiol. Dis..

[104] Long A.N., Owens K., Schlappal A.E., Kristian T., Fishman P.S., Schuh R.A. (2015). Effect of nicotinamide mononucleotide on brain mitochondrial respiratory deficits in an Alzheimer’s disease-relevant murine model. BMC Neurol.

[105] Fukuwatari T., Shibata K., Ishihara K., Fushiki T., Sugimoto E. (2001). Elevation of blood NAD level after moderate exercise in young women and mice. J. Nutr. Sci. Vitaminol. (Tokyo).

[106] Wen D.T., Zheng L., Li J.X., Cheng D., Liu Y., Lu K. (2019). Endurance exercise resistance to lipotoxic cardiomyopathy is associated with cardiac NAD^+^/dSIR2/PGC-1 alpha pathway activation in old Drosophila. Biol. Open.

[107] Wei X.J., Jia R., Wang G., Hong S.Y., Song L., Sun B. (2020). Depot-specific regulation of NAD^+^/SIRTs metabolism identified in adipose tissue of mice in response to high-fat diet feeding or calorie restriction. J. Nutr. Biochem..

[108] Moroz N., Carmona J.J., Anderson E., Hart A.C., Sinclair D.A., Blackwell T.K. (2014). Dietary restriction involves NAD^+^-dependent mechanisms and a shift toward oxidative metabolism. Aging Cell.

[109] Levine D.C., Hong H., Weidemann B.J., Ramsey K.M., Affinati A.H., Schmidt M.S. (2020). NAD^+^ controls circadian reprogramming through PER2 nuclear translocation to counter aging. Mol. Cell.

[110] Sahar S., Nin V., Barbosa M.T., Chini E.N., Sassone-Corsi P. (2011). Altered behavioral and metabolic circadian rhythms in mice with disrupted NAD^+^ oscillation. Aging US.

[111] Benzi A., Sturla L., Heine M., Fischer A.W., Spinelli S., Magnone M. (2021). CD38 downregulation modulates NAD^+^ and NADP(H) levels in thermogenic adipose tissues. Biochim. Biophys. Acta Mol. Cell Biol. Lipids.

[112] Roboon J., Hattori T., Ishii H., Takarada-Iemata M., Nguyen D.T., Heer C.D. (2021). Inhibition of CD38 and supplementation of nicotinamide riboside ameliorate lipopolysaccharide-induced microglial and astrocytic neuroinflammation by increasing NAD. J. Neurochem..

[113] Almeida G.S., Bawn C.M., Galler M., Wilson I., Thomas H.D., Kyle S. (2017). PARP inhibitor rucaparib induces changes in NAD levels in cells and liver tissues as assessed by MRS. NMR Biomed.

[114] Pinkerton A.B., Sessions E.H., Hershberger P., Maloney P.R., Peddibhotla S., Hopf M. (2021). Optimization of a urea-containing series of nicotinamide phosphoribosyltransferase (NAMPT) activators. Bioorg. Med. Chem. Lett..

[115] Zhang N., Sauve A.A. (2018). Regulatory effects of NAD^+^ metabolic pathways on sirtuin activity. Prog. Mol. Biol. Transl. Sci..

[116] Wang G., Han T., Nijhawan D., Theodoropoulos P., Naidoo J., Yadavalli S. (2014). P7C3 neuroprotective chemicals function by activating the rate-limiting enzyme in NAD salvage. Cell.

[117] Grant R., Berg J., Mestayer R., Braidy N., Bennett J., Broom S. (2019). A pilot study investigating changes in the human plasma and urine NAD^+^ metabolome during a 6 hour intravenous infusion of NAD. Front. Aging Neurosci..

[118] Kim M., Seol J., Sato T., Fukamizu Y., Sakurai T., Okura T. (2022). Effect of 12-week intake of nicotinamide mononucleotide on sleep quality, fatigue, and physical performance in older Japanese adults: a randomized, double-blind placebo-controlled study. Nutrients.

[119] Brakedal B., Dölle C., Riemer F., Ma Y.L., Nido G.S., Skeie G.O. (2022). The NADPARK study: a randomized phase I trial of nicotinamide riboside supplementation in Parkinson’s disease. Cell Metab.

[120] Martens C.R., Denman B.A., Mazzo M.R., Armstrong M.L., Reisdorph N., McQueen M.B. (2018). Chronic nicotinamide riboside supplementation is well-tolerated and elevates NAD^+^ in healthy middle-aged and older adults. Nat. Commun..

[121] Connell N.J., Grevendonk L., Fealy C.E., Moonen-Kornips E., Bruls Y.M.H., Schrauwen-Hinderling V.B. (2021). NAD^+^-precursor supplementation with l-tryptophan, nicotinic acid, and nicotinamide does not affect mitochondrial function or skeletal muscle function in physically compromised older adults. J. Nutr..

[122] Pencina K.M., Lavu S., dos Santos M., Beleva Y.M., Cheng M., Livingston D. (2023). MIB-626, an oral formulation of a microcrystalline unique polymorph of β-nicotinamide mononucleotide, increases circulating nicotinamide adenine dinucleotide and its metabolome in middle-aged and older adults. J. Gerontol. A Biol. Sci. Med. Sci..

[123] Zapata-Perez R., Wanders R.J.A., van Karnebeek C.D.M., Houtkooper R.H. (2021). NAD^+^ homeostasis in human health and disease. EMBO Mol. Med..

[124] Tran T., Pencina K.M., Schultz M.B., Li Z.Y., Ghattas C., Lau J. (2022). Reduced levels of NAD in skeletal muscle and increased physiologic frailty are associated with viral coinfection in asymptomatic middle-aged adults. J. Acquir. Immune Defic. Syndr..

[125] Yang F., Zhang X.G., Hu F.F., Yu Y., Luo L., Deng X. (2022). Association between NAD^+^ levels and anaemia among women in community-based study. J. Cell. Mol. Med..

[126] Bai X.Y., Wang P. (2022). Relationship between sperm NAD^+^ concentration and reproductive aging in normozoospermia men: a cohort study. BMC Urol.

[127] Xiao N., Nie M., Pang H.H., Wang B.H., Hu J.L., Meng X.J. (2021). Integrated cytokine and metabolite analysis reveals immunometabolic reprogramming in COVID-19 patients with therapeutic implications. Nat. Commun..

[128] Liu L., Su X., Quinn W.J., Hui S., Krukenberg K., Frederick D.W. (2018). Quantitative analysis of NAD synthesis-breakdown fluxes. Cell Metab.

[129] Yiasemides E., Sivapirabu G., Halliday G.M., Park J., Damian D.L. (2009). Oral nicotinamide protects against ultraviolet radiation-induced immunosuppression in humans. Carcinogenesis.

[130] Trammell S.A.J., Schmidt M.S., Weidemann B.J., Redpath P., Jaksch F., Dellinger R.W. (2016). Nicotinamide riboside is uniquely and orally bioavailable in mice and humans. Nat. Commun..

[131] Elhassan Y.S., Kluckova K., Fletcher R.S., Schmidt M.S., Garten A., Doig C.L. (2019). Nicotinamide riboside augments the aged human skeletalmuscle NAD^+^ metabolome and induces transcriptomic and anti-inflammatory signatures. Cell Rep.

[132] Airhart S.E., Shireman L.M., Risler L.J., Anderson G.D., Nagana Gowda G.A., Raftery D. (2017). An open-label, non-randomized study of the pharmacokinetics of the nutritional supplement nicotinamide riboside (NR) and its effects on blood NAD^+^ levels in healthy volunteers. PLOS ONE.

[133] Zhou B., Wang D.D., Qiu Y., Airhart S., Liu Y., Stempien-Otero A. (2020). Boosting NAD level suppresses inflammatory activation of PBMCs in heart failure. J. Clin. Invest..

[134] Wu J., Singh K., Lin A., Meadows A.M., Wu K., Shing V. (2022). Boosting NAD^+^ blunts TLR4-induced type I IFN in control and systemic lupus erythematosus monocytes. J. Clin. Invest..

[135] Wang D.D., Airhart S.E., Zhou B., Shireman L.M., Jiang S., Melendez Rodriguez C. (2022). Safety and tolerability of nicotinamide riboside in heart failure with reduced ejection fraction, JACC Basic Transl. Sci.

[136] Vreones M., Mustapic M., Moaddel R., Pucha K.A., Lovett J., Seals D.R. (2023). Oral nicotinamide riboside raises NAD+ and lowers biomarkers of neurodegenerative pathology in plasma extracellular vesicles enriched for neuronal origin. Aging Cell.

[137] Lapatto H.A.K., Kuusela M., Heikkinen A., Muniandy M., van der Kolk B.W., Gopalakrishnan S. (2023). Nicotinamide riboside improves muscle mitochondrial biogenesis, satellite cell differentiation, and gut microbiota in a twin study. Sci. Adv..

[138] Conze D., Brenner C., Kruger C.L. (2019). Safety and metabolism of long-term administration of NIAGEN (nicotinamide riboside chloride) in a randomized, double-blind, placebo-controlled clinical trial of healthy overweight adults. Sci. Rep..

[139] Remie C.M.E., Roumans K.H.M., Moonen M.P.B., Connell N.J., Havekes B., Mevenkamp J. (2020). Nicotinamide riboside supplementation alters body composition and skeletal muscle acetylcarnitine concentrations in healthy obese humans. Am. J. Clin. Nutr..

[140] Okabe K., Yaku K., Uchida Y., Fukamizu Y., Sato T., Sakurai T. (2022). Oral administration of nicotinamide mononucleotide is safe and efficiently increases blood nicotinamide adenine dinucleotide levels in healthy subjects. Front. Nutr..

[141] Igarashi M., Nakagawa-Nagahama Y., Miura M., Kashiwabara K., Yaku K., Sawada M. (2022). Chronic nicotinamide mononucleotide supplementation elevates blood nicotinamide adenine dinucleotide levels and alters muscle function in healthy older men. NPJ Aging.

[142] Dollerup O.L., Chubanava S., Agerholm M., Søndergård S.D., Altıntaş A., Møller A.B. (2020). Nicotinamide riboside does not alter mitochondrial respiration, content or morphology in skeletal muscle from obese and insulin-resistant men. J. Physiol..

[143] Dollerup O.L., Christensen B., Svart M., Schmidt M.S., Sulek K., Ringgaard S. (2018). A randomized placebo-controlled clinical trial of nicotinamide riboside in obese men: safety, insulin-sensitivity, and lipid-mobilizing effects. Am. J. Clin. Nutr..

[144] Dolopikou C.F., Kourtzidis I.A., Margaritelis N.V., Vrabas I.S., Koidou I., Kyparos A. (2020). Acute nicotinamide riboside supplementation improves redox homeostasis and exercise performance in old individuals: a double-blind cross-over study. Eur. J. Nutr..

[145] Liao B.G., Zhao Y.L., Wang D., Zhang X.W., Hao X.M., Hu M. (2021). Nicotinamide mononucleotide supplementation enhances aerobic capacity in amateur runners: a randomized, double-blind study. J. Int. Soc. Sports Nutr..

[146] Huang H. (2022). A multicentre, randomised, double blind, parallel design, placebo controlled study to evaluate the efficacy and safety of uthever (NMN supplement), an orally administered supplementation in middle aged and older adults. Front. Aging.

[147] Niu K.M., Bao T.T., Gao L.M., Ru M., Li Y.M., Jiang L. (2021). The impacts of short-term NMN supplementation on serum metabolism, fecal microbiota, and telomere length in pre-aging phase. Front. Nutr..

[148] Kei A., Elisaf M.S. (2012). Nicotinic acid: clinical considerations. Expert Opin. Drug Saf..

[149] Snaidr V.A., Damian D.L., Halliday G.M. (2019). Nicotinamide for photoprotection and skin cancer chemoprevention: a review of efficacy and safety. Exp. Dermatol..

[150] Lenglet A., Liabeuf S., El Esper N., Brisset S., Mansour J., Lemaire-Hurtel A.S. (2017). Efficacy and safety of nicotinamide in haemodialysis patients: the NICOREN study. Nephrol. Dial, Transplant..

[151] Gale E.A., Bingley P.J., Emmett C.L., Collier T. (2004). European Nicotinamide Diabetes Intervention Trial (ENDIT) Group, European Nicotinamide Diabetes Intervention Trial (ENDIT): a randomised controlled trial of intervention before the onset of type 1 diabetes. Lancet.

[152] El-Kady R.R., Ali A.K., El Wakeel L.M., Sabri N.A., Shawki M.A. (2022). Nicotinamide supplementation in diabetic nonalcoholic fatty liver disease patients: randomized controlled trial. Ther. Adv. Chronic Dis..

[153] Braidy N., Liu Y. (2020). NAD^+^ therapy in age-related degenerative disorders: a benefit/risk analysis. Exp. Gerontol..

[154] Kawamura T., Mori N., Shibata K. (2016). β-nicotinamide mononucleotide, an anti-aging candidate compound, is retained in the body for longer than nicotinamide in rats. J. Nutr. Sci. Vitaminol. (Tokyo).

[155] Bogan K.L., Brenner C. (2008). Nicotinic acid nicotinamide and nicotinamide riboside: a molecular evaluation of NAD^+^ precursor vitamins in human nutrition. Annu. Rev. Nutr..

[156] Yoshino J., Baur J.A., Imai S.I. (2018). NAD^+^ intermediates: the biology and therapeutic potential of NMN and NR. Cell Metab.

[157] Sharma C., Donu D., Cen Y.N. (2022). Emerging role of nicotinamide riboside in health and diseases. Nutrients.

[158] Mehmel M., Jovanović N., Spitz U. (2020). Nicotinamide riboside-the current state of research and therapeutic uses. Nutrients.

[159] Bitterman K.J., Anderson R.M., Cohen H.Y., Latorre-Esteves M., Sinclair D.A. (2002). Inhibition of silencing and accelerated aging by nicotinamide, a putative negative regulator of yeast sir2 and human SIRT1. J. Biol. Chem..

[160] Grozio A., Mills K.F., Yoshino J., Bruzzone S., Sociali G., Tokizane K. (2019). Slc12a8 is a nicotinamide mononucleotide transporter. Nat. Metab..

[161] Schmidt M.S., Brenner C. (2019). Absence of evidence that Slc12a8 encodes a nicotinamide mononucleotide transporter. Nat. Metab..

[162] Ratajczak J., Joffraud M., Trammell S.A.J., Ras R., Canela N., Boutant M. (2016). NRK1 controls nicotinamide mononucleotide and nicotinamide riboside metabolism in mammalian cells. Nat. Commun..

[163] Mateuszuk Ł., Campagna R., Kutryb-Zając B., Kuś K., Słominska E.M., Smolenski R.T. (2020). Reversal of endothelial dysfunction by nicotinamide mononucleotide via extracellular conversion to nicotinamide riboside. Biochem. Pharmacol..

[164] Fletcher R.S., Ratajczak J., Doig C.L., Oakey L.A., Callingham R., Xavier G.D. (2017). Nicotinamide riboside kinases display redundancy in mediating nicotinamide mononucleotide and nicotinamide riboside metabolism in skeletal muscle cells. Mol. Metab..

[165] Fragola G., Mabb A.M., Taylor-Blake B., Niehaus J.K., Chronister W.D., Mao H.Q. (2020). Deletion of topoisomerase 1 in excitatory neurons causes genomic instability and early onset neurodegeneration. Nat. Commun..

[166] Kropotov A., Kulikova V., Nerinovski K., Yakimov A., Svetlova M., Solovjeva L. (2021). Equilibrative nucleoside transporters mediate the import of nicotinamide riboside and nicotinic acid riboside into human cells. Int. J. Mol. Sci..

[167] Kim L.J., Chalmers T.J., Madawala R., Smith G.C., Li C., Das A. (2023). Host-microbiome interactions in nicotinamide mononucleotide (NMN) deamidation. FEBS Lett.

[168] (2022). PubChem Compound Summary for CID 14180, Nicotinamide mononucleotide.

[169] Liu X.F., Jiang Y.Y., Wang C., Li X.W., Yang Z.G., Leng K. (2021). Determination of nicotinamide mononucleotide in the natural food materials by high performance liquid chromatography-mass spectrometry. Food Sci. Technol..

[170] Dietary Reference Intakes for Thiamin (1998).

[171] Centers for Disease Control (CDC) (1983). Niacin intoxication from pumpernickel bagels - New York. MMWR Morb. Mortal. Wkly. Rep..

[172] Rolfe H.M. (2014). A review of nicotinamide: treatment of skin diseases and potential side effects. J. Cosmet. Dermatol..

[173] Prousky J., Seely D. (2005). The treatment of migraines and tension-type headaches with intravenous and oral niacin (nicotinic acid): systematic review of the literature. Nutr. J..

[174] Schwartz M.L. (1993). Severe reversible hyperglycemia as a consequence of niacin therapy. Arch. Intern. Med..

[175] Fraunfelder F.W., Fraunfelder F.T., Illingworth D.R. (1995). Adverse ocular effects associated with niacin therapy. Br. J. Ophthalmol..

[176] Rader J.I., Calvert R.J., Hathcock J.N. (1992). Hepatic toxicity of unmodified and time-release preparations of niacin. Am. J. Med..

[177] Bieganowski P., Brenner C. (2004). Discoveries of nicotinamide riboside as a nutrient and conserved NRK genes establish a preiss-handler independent route to NAD^+^ in fungi and humans. Cell.

[178] Fukamizu Y., Uchida Y., Shigekawa A., Sato T., Kosaka H., Sakurai T. (2022). Safety evaluation of beta-nicotinamide mononucleotide oral administration in healthy adult men and women. Sci. Rep..

[179] Institute of Medicine, Otten J.J., Hellwig J.P., Meyers L.D. (2006). Dietary Reference Intakes: The Essential Guide to Nutrient Requirements.

[180] Yang F., Deng X., Yu Y., Luo L., Chen X.D., Zheng J.P. (2022). Association of human whole blood NAD^+^ contents with aging. Front. Endocrinol..

[181] (2014). European Food Safety Authority (EFSA). Scientific opinion on dietary reference values for niacin, EFSA J..

[182] Bai L.B., Yau L.F., Tong T.T., Chan W.H., Zhang W., Jiang Z.H. (2022). Improvement of tissue-specific distribution and biotransformation potential of nicotinamide mononucleotide in combination with ginsenosides or resveratrol. Pharmacol. Res. Perspect..

[183] Black W.B., Aspacio D., Bever D., King E., Zhang L.Y., Li H. (2020). Metabolic engineering of Escherichia coli for optimized biosynthesis of nicotinamide mononucleotide, a noncanonical redox cofactor. Microb. Cell Fact..

[184] Shoji S., Yamaji T., Makino H., Ishii J., Kondo A. (2021). Metabolic design for selective production of nicotinamide mononucleotide from glucose and nicotinamide. Metab. Eng..

[185] Li Q.Z., Meng D.D., You C. (2023). An artificial multi-enzyme cascade biocatalysis for biomanufacturing of nicotinamide mononucleotide from starch and nicotinamide in one-pot. Enzyme Microb. Technol..

[186] Lee J., Churchil H., Choi W.B., Lynch J.E., Roberts F.E., Volante R.P. (1999). A chemical synthesis of nicotinamide adenine dinucleotide (NAD^+^). Chem. Commun..

[187] Tanimori S., Ohta T., Kirihata M. (2002). An efficient chemical synthesis of nicotinamide riboside (NAR) and analogues. Bioorg. Med. Chem. Lett..

[188] Franchetti P., Pasqualini M., Petrelli R., Ricciutelli M., Vita P., Cappellacci L. (2004). Stereoselective synthesis of nicotinamide beta-riboside and nucleoside analogs. Bioorg. Med. Chem. Lett..

[189] Liu W.J., Wu S.G., Hou S.H., Zhao Z.B. (2009). Synthesis of phosphodiester-type nicotinamide adenine dinucleotide analogs. Tetrahedron.

[190] Shen Q., Zhang S.J., Xue Y.Z., Peng F., Cheng D.Y., Xue Y.P. (2021). Biological synthesis of nicotinamide mononucleotide. Biotechnol. Lett..

[191] Marinescu G.C., Popescu R.G., Stoian G., Dinischiotu A. (2018). Beta-nicotinamide mononucleotide (NMN) production in Escherichia coli. Sci. Rep..

[192] Maharjan A., Singhvi M., Kim B.S. (2021). Biosynthesis of a therapeutically important nicotinamide mononucleotide through a phosphoribosyl pyrophosphate synthetase 1 and 2 engineered strain of Escherichia coli. ACS Synth. Biol..

[193] Di Stefano M., Loreto A., Orsomando G., Mori V., Zamporlini F., Hulse R.P. (2017). NMN deamidase delays wallerian degeneration and rescues axonal defects caused by NMNAT2 deficiency in vivo. Curr. Biol..

[194] Di Stefano M., Nascimento-Ferreira I., Orsomando G., Mori V., Gilley J., Brown R. (2015). A rise in NAD precursor nicotinamide mononucleotide (NMN) after injury promotes axon degeneration. Cell Death Differ.

[195] Nacarelli T., Lau L., Fukumoto T., Zundell J., Fatkhutdinov N., Wu S. (2019). NAD^+^ metabolism governs the proinflammatory senescence-associated secretome. Nat. Cell Biol..

[196] Zhao Z.Y., Xie X.J., Li W.H., Liu J., Chen Z., Zhang B. (2019). A cell-permeant mimetic of NMN activates SARM1 to produce cyclic ADP-ribose and induce non-apoptotic cell death. iScience.

[197] Nakahata Y., Sahar S., Astarita G., Kaluzova M., Sassone-Corsi P. (2009). Circadian control of the NAD^+^ salvage pathway by CLOCK-SIRT1. Science.

[198] Ramsey K.M., Yoshino J., Brace C.S., Abrassart D., Kobayashi Y., Marcheva B. (2009). Circadian clock feedback cycle through NAMPT-mediated NAD^+^ biosynthesis. Science.

[199] Hackney A.C., Schumann M., Rønnestad B. (2018). Concurrent Aerobic and Strength Training.

[200] Brown K.D., Maqsood S., Huang J.Y., Pan Y., Harkcom W., Li W. (2014). Activation of SIRT3 by the NAD^+^ precursor nicotinamide riboside protects from noise-induced hearing loss. Cell Metab.

[201] Han S.G., Du Z.D., Liu K., Gong S.S. (2020). Nicotinamide riboside protects noise-induced hearing loss by recovering the hair cell ribbon synapses. Neurosci. Lett..

[202] Rauch B.H., Weber A., Braun M., Zimmerman N., Schrör K. (2000). PDGF-induced Akt phosphorylation does not activate NF-κB in human vascular smooth muscle cells and fibroblasts. FEBS Lett.

[203] Hoehn K.L., Hohnen-Behrens C., Cederberg A., Wu L.E., Turner N., Yuasa T. (2008). IRS1-independent defects define major nodes of insulin resistance. Cell Metab.

[204] von Zglinicki T., Martin-Ruiz C. (2005). Telomeres as biomarkers for ageing and age-related diseases. Curr. Mol. Med..

[205] Amano H., Chaudhury A., Rodriguez-Aguayo C., Lu L., Akhanov V., Catic A. (2019). Telomere dysfunction induces sirtuin repression that drives telomere-dependent disease. Cell Metab.

[206] Brito S., Baek J.M., Cha B., Heo H., Lee S.H., Lei L. (2022). Nicotinamide mononucleotide reduces melanin production in aged melanocytes by inhibiting cAMP/Wnt signaling. J. Dermatol. Sci..

