# Supplementary material for: The Safety and Antiaging Effects of Nicotinamide Mononucleotide in Human Clinical Trials: an Update
Source: Adv Nutr. 2023 Aug 22;14(6):1416–35. doi: 10.1016/j.advnut.2023.08.008 (PMC10721522; doi:10.1016/j.advnut.2023.08.008)
Supplement: Multimedia component 1 [file mmc1.docx]

**The Safety and Anti-Ageing Effects of Nicotinamide Mononucleotide in Human Clinical Trials: An Update**

Qin Song^1#^, Xiaofeng Zhou^2#^, Kexin Xu^3^, Sishi Liu^3^, Xinqiang Zhu^4^*, and Jun Yang^3,5^*

**Supplementary table 1** The effects of NAD**^+^** precursors on the levels of NAD**^+^** and its metabolites in human clinical trials

| **Dose** | **Duration** | **NAD^+^** | **NAD^+^ metabolome** | **Test sample** | **References** |
| --- | --- | --- | --- | --- | --- |
| NMN 250 mg/day | 5 hours | - | ↑ 2Py, ↑ 4Py | Plasma | (35) |
| NMN 500 mg/day | 5 hours | - | ↑ 2Py, ↑ 4Py | Plasma |  |
| Baseline (Placebo) | 2 weeks | No specific value is given | NMN 32.6 ng/mL, NAM 10.2 ng/mL, 1-Methyl NAM 7.57 ng/mL, 2-Py-NAM 103 ng/mL, NR 0.406 ng/mL | Blood | (122) |
| NMN 1 g once daily | 2 weeks | ↑ Increasing about 1-folds | ↑ NMN 88.2 ng/mL, ↑ NAM 65.2 ng/mL, ↑ 1-Methyl NAM 146 ng/mL, ↑ 2-Py-NAM 2150 ng/mL, ↑ NR 1.3 ng/mL | Blood |  |
| NMN 1 g twice daily | 2 weeks | ↑ Increasing about 2-folds | ↑ NMN 148 ng/mL, ↑ NAM 140 ng/mL, ↑ 1-Methyl NAM 276 ng/mL, ↑ 2-Py-NAM 4230 ng/mL, ↑ NR 1.48 ng/mL | Blood |  |
| Baseline (Placebo) | 2 weeks | - | ↑ 2Py 16700 ng/mL  No significant change: NMN 333 ng/mL, NAM 174 ng/mL | Urine |  |
| NMN 1 g once daily | 2 weeks | - | ↑ 2Py 137000  No significant change: NMN 384 ng/mL, NAM 290 ng/mL | Urine |  |
| NMN 1 g twice daily | 2 weeks | - | ↑ 2Py 273000 ng/mL  No significant change: NMN 308 ng/mL, NAM 703 ng/mL | Urine |  |
| Baseline (Placebo) | 4/8/12/16 weeks | About 20 μm | No specific value is given | Blood | (140) |
| NMN 250 mg/day | 4 weeks | ↑ Increasing about 2.5-folds | ↑ NAMN  No significant change: NAAD, NMN, NR, NAR, NAM, NA, MNAM | Blood |  |
| NMN 250 mg/day | 8 weeks | ↑ Increasing about 2-folds | ↑ NAMN  No significant change: NAAD, NMN, NR, NAR, NAM, NA, MNAM | Blood |  |
| NMN 250 mg/day | 12 weeks | ↑ Increasing about 1.7-folds | ↑ NAMN  No significant change: NAAD, NMN, NR, NAR, NAM, NA, MNAM | Blood |  |
| NMN 250 mg/day | 16 weeks | No significant change | No significant change: NAMN, NAAD, NMN, NR, NAR, NAM, NA, MNAM | Blood |  |
| Baseline (Placebo) | 10 weeks | About 30 pg/mg | No specific value is given | PBMCs/ Plasma | (36) |
| NMN 250 mg/day | 10 weeks | ↑ Increasing about 1.7-folds in PBMC | ↑ 2Py and 4Py in plasma | PBMCs/ Plasma |  |
| Baseline (Placebo) | 10 weeks | About 3.5 pg/mg | No specific value is given | Skeletal muscle |  |
| NMN 250 mg/day | 10 weeks | No significant change | ↑ 2Py and 4Py | Skeletal muscle |  |
| Baseline (Placebo) | 12 weeks | About 0.35 μm | No specific value is given | Blood | (141) |
| NMN 250 mg/day | 12 weeks | ↑ Increasing about 2.57-folds | ↑ NMN, ↑ NR, ↑ NAMN, ↑ NAR  No significant change: NA, NAM | Blood |  |
| Baseline (Placebo) | 3 weeks | 210 pmol/mg | NAAD 0.35 pmol/mg, MeNAM 0.35 pmol/mg,  Me-2Py 1.1 pmol/mg, Me-4Py 0.3 pmol/mg, NR 1.25 pmol/mg, NAM 86.5 pmol/mg | Skeletal muscle | (131) |
| NR 1g/day | 3 weeks | 197 pmol/mg | ↑ NAAD 0.73 pmol/mg, ↑ MeNAM 1.45 pmol/mg, ↑ Me-2-Py 6.6 pmol/mg, ↑ Me-4Py 1.6 pmol/mg  No significant change: NR 1.4 pmol/mg, NAM 92 pmol/mg | Skeletal muscle |  |
| Baseline (Placebo) | 3 weeks | 20.9 μm | NMN 1.13 μm, NAAD 0.04μm, MeNAM 0.1μm, Me-2Py 1.44μm, Me-4Py 0.48μm, NR 0.15μm, NAM 9.5μm | Blood |  |
| NR 1g/day | 3 weeks | ↑ 47.75 μm | ↑ NMN 1.63 μm, ↑ NAAD 0.18 μm, ↑ MeNAM 0.66 μm, ↑ Me-2Py 7.69 μm, ↑ Me-4Py 3.82 μm  No significant change: NR 0.16 μm, NAM 10.6 μm | Blood |  |
| Baseline (Placebo) | 3 weeks | - | NAR 10.3 μmol/mol, NR 31.7 μmol/mol, NAM 106.5 μmol/mol | Urine |  |
| NR 1g/day | 3 weeks | - | ↑ Me-2-py, ↑ Me-4Py*,* ↑ NAR 185.5 μmol/mol, ↑ NAM 282μmol/mol  No significant change: NR 41.5 μmol/mol | Urine |  |
| NR 1 g twice daily | 12 weeks | - | ↑ NAM  No significant change: MeNAM, Me-2Py, Me-4Py, NAR | Urine | (143) |
| NR 500 mg/day | 2 days | - | ↑ NADH, ↑ NADPH | Erythrocytes | (144) |
| Baseline (Placebo) | 6 weeks | 6.2 pmol/mg | NADP 3.3−17.9 pmol/mg, NAM 109−411 pmol/mg, NAAD 0.0−2.3 pmol/mg, NMN 0.0−5.5 pmol/mg | PBMCs | (120) |
| NR 1000 mg/day | 6 weeks | ↑ Increasing about 1.6-folds | ↑ NAAD 0.0−8.7  No significant change: NADP 2.7−42.7 pmol/mg, NAM 171−1357 pmol/mg, pmol/mg, NMN 0.0−11.9 pmol/mg | PBMCs |  |
| Baseline (Placebo) | 12 weeks | 500-2500 pmol/mg | NADH 100-1000 pmol/mg, NADP 50-400 pmol/mg, NADPH 100-500 pmol/mg | Skeletal muscle | (142) |
| NR 1000 mg/day | 12 weeks | No significant change | All no significant changes | Skeletal muscle |  |
| NR 1 g/day | 1 weeks | ↑ Increasing 2.7-folds | ↑ NMN, ↑ NADP, ↑ NAM, ↑ MeNAM, ↑ Me-2Py, ↑ Me-4Py, ↑ NAAD | PBMCs | (130) |
| NR 1 g/day | 1 weeks | - | ↑ NAM, ↑ MeNAM, ↑ Me-2Py, ↑ Me-4Py | Plasma |  |
| NR 1 g/day | 1 weeks | - | ↑ NAM, ↑ MeNAM, ↑ Me-2Py, ↑ Me-4Py | Urine |  |
| NAM 500 mg | 1 hour | ↑ Increasing about 1.3-folds | - | Blood | (129) |
| NAM 500 mg | 1.5 hours | No significant change | - | Blood |  |
| Baseline | 9 days | 27 μM | - | Blood | (132) |
| NR 250-2000 mg/day | 9 days | ↑ 50 μM | - | Blood |  |
| Baseline (Placebo) | 8 weeks | 21.0 ng/ml | NAM 22.3 ng/ml, MeNAM 3.1 ng/ml | Blood | (138) |
| NR 100 mg /day | 8 weeks | 24.3 ng/ml | No significant change: NAM 26.6 ng/ml, MeNAM 5.6 ng/ml | Blood |  |
| NR 300 mg /day | 8 weeks | ↑ 32.3 ng/ml | ↑ MeNAM 10.1 ng/ml, No significant change: NAM 27.9 ng/ml | Blood |  |
| NR 1000 mg /day | 8 weeks | ↑ 49.2 ng/ml | ↑ NAM 43.7 ng/ml, ↑ MeNAM 26.6 ng/ml | Blood |  |
| Baseline (Placebo) | 8 weeks | - | MeNAM 4.1 ng/μg creatinine, Me2PY 15 ng/μg creatinine | Urine |  |
| NR 100 mg /day | 8 weeks | - | No significant change: MeNAM 6.6 ng/μg creatinine, Me2PY 30 ng/μg creatinine | Urine |  |
| NR 300 mg /day | 8 weeks | - | ↑ MeNAM 10.6 ng/μg creatinine, ↑ Me2PY 51 ng/μg creatinine | Urine |  |
| NR 1000 mg /day | 8 weeks | - | ↑ MeNAM 17.8 ng/μg creatinine, ↑ Me2PY 113 ng/μg creatinine | Urine |  |
| Baseline (Placebo) | 6 weeks | 1.019 nmol/mg | NAAD, NAD, NADH, NADP, NADPH, NAM, NMN, MeNAM | Skeletal muscle | (139) |
| NR 1000 mg /day | 6 weeks | 1.125 nmol/mg | ↑ NAAD, ↑ MeNAM, No significant change: NAD, NADH, NADP, NADPH, NAM, NMN | Skeletal muscle |  |
| Baseline | 9 days | No specific value is given | - | Blood | (133) |
| NR 2000 mg/day | 9 days | ↑ Increasing about 2-folds | - | Blood |  |
| Baseline (Placebo) | 7 days | No specific value is given | No specific value is given | Blood | (134) |
| NR 1000 mg/day | 7 days | ↑ Increasing about 2-folds | ↑ NAAD, ↑ ADPR, ↑ Me-4Py | Blood |  |
| Baseline (Placebo) | 32.4 ±2.53 days | No specific value is given | No specific value is given | Cerebrospinal fluid | (119) |
| NR 1000 mg/day | 32.5 ±2.7 days | - | ↑ Me2PY | Cerebrospinal fluid |  |
| NR 1000 mg/day | 32.5 ±2.7 days | - | ↑ NAAD, ↑ MeNAM, ↑ Me2PY, ↑ Me-4Py, ↑ Nam N-oxide | Skeletal muscle |  |
| NR 1000 mg/day | 32.5 ±2.7 days | - | ↑ NAAD, ↑ MeNAM | PBMCs |  |
| Baseline (Placebo) | 10 weeks | No specific value is given | - | Blood | (135) |
| NR 500-1000 mg/day | 10 weeks | ↑ Increasing about 2-folds | - | Blood |  |
| Baseline (Placebo) | 6 weeks | No specific value is given | No specific value is given | Blood | (136) |
| NR 500 mg twice daily | 6 weeks | ↑ Increasing about 1-folds | No significant change: NADH | Blood |  |
| Baseline | 5 months | No specific value is given | No specific value is given | Blood | (137) |
| NR 250 mg/week, then 1000 mg/day | 1 month, then 4 months | ↑ Increasing about 2-folds | ↑ NAAD, ↑ NMN, ↑ Me-4Py, No significant change: NAR, NADP, ADPR | Blood |  |

“-”：It was not been tested, “↑”: Significant increasing.

**Supplementary table 2** Completed but unpublished clinical trials and ongoing clinical trials

| **The safety and metabolic kinetics of NMN** | | | | | | | | |
| --- | --- | --- | --- | --- | --- | --- | --- | --- |
| **Trial Name** | **Registration number** | | **Design** | **Dose & duration** | **Indicators** | **Status** | **Location** | **Registration time** |
| Assessment of the safety of long-term nicotinamide mononucleotide (NMN) | UMIN000030609 | | Open label, non-randomized, uncontrolled study; 30 healthy male aged 40-60 years | Oral administration: intake of NMN for 8 weeks | Physical and laboratory examinations, the kinetics of NMN and metabolites of nicotinamide, the effect of daily NMN administration on glucose metabolism | Completed | Japan | 2019 |
| Safety assessment of the yeast extract containing nicotinamide mononucleotide (NMN) | UMIN000039527 | Double-blind, randomized, placebo-controlled study; 33 healthy adults aged 20-65 years | | Oral administration: 2.5 g/day for 12 weeks | Height, weight, body fat percentage, BMI, systolic blood pressure, diastolic blood pressure, heart rate, blood test, urine testing, diary | Completed | Japan | 2019 |
| To evaluate the efficacy and safety of vitamin NMN in middle aged and older adults for anti-ageing and work-out enhancer | CTRI/2019/12/022514 | | Double-blind, randomized, placebo- controlled study; 66 adults aged 40-65 years | Oral administration: 2 capsules/day for 60 days | the efficacy of NMN in terms of stimulation of NAD^+^ metabolism | Completed | India | 2020 |
| A verification study of safety evaluation of excessive ingestion of NMN-containing food in humans: a randomized, double-blind, placebo-controlled, parallel study | UMIN000043084 | | Double-blind, randomized, placebo-controlled study; 32 adults aged 20 - 65 years | Oral administration: NMN-containing food once a day for 4 weeks. | Physical examination, urinalysis, blood test | Completed | Japan | 2021 |
| Safety observation study for healthy individuals of intravenous administration of NMN | UMIN000047134 | | Open label, non-randomized, uncontrolled study; 10 healthy adults aged 20-70 years | Single intravenous administration of NMN | NAD^+^ levels and SIRT1 activation in blood | Completed | Japan | 2021 |
| To evaluate the efficacy and safety of NMN as an anti-ageing Supplement in middle aged and older (40-65 years) adults | NCT04823260 | | Double-blind, randomized, placebo-controlled study; 90 healthy adults aged 40-65 years | Oral administration: 300 mg/day and 600 mg/day for 60 days | Blood cellular NAD+/ NADH, six walking endurance test, SF-36 questionnaire, telomerase test results, BMI, biological age using ageing. Ai 3.0 calculator | Completed | India | 2021 |
| Safety and pharmacokinetics of nicotinamide mononucleotide (NMN) in healthy adults | NCT04910061 | | Open label, non-randomized, non-controlled, monocentre repeated-dose study; 24 healthy adults aged 18-65 years | Oral administration:400 mg/day for 29 days | Adverse events, body temperature, heart rate and blood pressure, complete blood count, C reactive protein, AST, ALT, bilirubin, GGT, alkaline phosphatase, creatinine, creatine kinase, sodium, potassium, chloride | Active, not recruiting | Canada | 2021 |
| Pharmacodynamics and tolerance of nicotinamide mononucleotide (NMN) in healthy adults | NCT04862338 | | Open-label, single-arm, single-center study; 20 healthy adults aged 30-60 years | Oral administration:400 mg/day for 28 days | NAD^+^ and NMN concentrations in whole blood, NAD^+^ metabolite concentrations in plasma and urine, adverse events, mitochondrial DNA ratio, blood glucose and lipid levels, transaminases (ASAT, ALAT, GGT) levels in blood, blood cell count; bilirubin, creatinine, and CPK levels in blood, blood ionogram, diastolic and systolic blood pressure, heart rate, weight, body composition | Active, not recruiting | France | 2021 |
| Effect of oral ingestion of NMN on blood NAD derivatives concentration | UMIN000047042 | | Open label, non-randomized, self-control study; 10 healthy adults aged 20-70 years | Oral administration:250 mg/day for 12 weeks | Blood NAD^+^ level | No longer recruiting | Japan | 2022 |
| **The efficacy of NMN on different diseases** | | | | | | | | |
| **Trial Name** | **Registration number** | | **Design** | **Dose and duration** | **Indicators** | **Status** | **Location** | **Registration time** |
| Impact of NMN for diabetic patients with physical frailty-NMN clinical study | jRCTs051190002 | | Double-blind, randomized, placebo-controlled study; 16 diabetic patients aged ≥ 65 years | Oral administration:250mg for six months | Grip strength and walking speed, muscle strength and volume, physical function, frality measurement (J-CHS scale), homed blood pressure and pulse rate measurement, diabetic condition and nephropathy, diabetic retinopathy | Completed | Japan | 2019 |
| Exploratory study of anti-aging effects of supplement intake | UMIN000043598 | | Double-blind, randomized, placebo-controlled study; 60 patients aged 45-65 years | Oral administration:2 capsules of NMN-containing continuously for 4 weeks | Mitochondrial number (mtDNA / gDNA), sirt1 gene expression level (sirt1 mRNA / GAPDH mRNA); urinary 8-OHdG, urinary pentosidine, blood fatty acid fraction, blood e-NAMPT, saliva Sirt2, intestinal flora, oral flora | Completed | Japan | 2021 |
| Effect of exercise nutrition intervention on pre-diabetes patients | ChiCTR2000040222 | | Parallel, placebo- controlled study; 100 [pre-diabetes patients](https://trialsearch.who.int/Trial2.aspx?TrialID=ChiCTR2000040222) aged 50-80 years | Oral administration:500 or 1000 mg/day | Complete biochemical examination, scale (Life style assessment scale, SF-36, SAS, SDS, GAD-7, PHQ-9), physical fitness (6-minute walk test, short physical performance battery, the Scale of ageing vigor in epidemiology), general information (height, weight, body composition, waist circumference, hip circumference, thigh circumference and blood pressure), noninvasive detection of vascular endothelial function | Recruiting | China | 2020 |
| Nicotinamide Mononucleotide in hypertensive patients | NCT04903210 | | Single-blind, randomized, parallel study; 20 hypertensive patients aged 18-65 years | Oral administration:400 mg/day for two months | Flow mediated dilation (FMD), brachial-ankle pulse wave velocity, blood pressure, PBMC NAD^+^ levels, sleep quality, adverse events | Recruiting | China | 2021 |
| Effect of NMN (nicotinamide mononucleotide) on polycystic ovary syndrome | NCT05305677 | | Double-blind, randomized, placebo-controlled study; 120 female patients aged 20-40 years | Oral administration:600mg/day for 8 weeks | Gut microbiota, metabolomics, glucose tolerance, homeostasis Model Assessment for Insulin Resistance (HOMA-IR) index, endocrine hormones, ovarian volume, follicle number, changes in blood NAD^+^ level, changes in BMI, changes in waist-to-hip ratio, changes in blood pressure | Recruiting | China | 2022 |
| Effect of NMN (nicotinamide mononucleotide) on diminished ovarian reserve (including premature ovarian insufficiency) | NCT05485610 | | Double-blind, randomized, placebo-controlled study; 220 female patients aged 20-40 years | Oral administration:600mg/day for 3 months | Gut microbiota composition, blood sugar level, fasting insulin, Endocrine hormones including AMH, ovarian volume, follicle number, blood NAD^+^ level, changes in NAD-related metabolites in urine | Not yet recruiting | China | 2022 |
| The anti-ageing effects of NMN on skin | | | | | | | | |
| Trial Name | Registration number | | Design | Dose and duration | Indicators | Status | Location | Registration time |
| Anti-wrinkle effect of beauty essence containing human stem cell components and NMN: single blinded study | UMIN000042828 | | Single-blinded, randomized study; 12 healthy female aged 35-59 years | External application: Only one side of the face, using test article for 4 consecutive weeks | Wrinkle grade | Completed | Japan | 2020 |
| Anti-ageing efficacy of a cosmetic formulation containing NMN (2%) versus placebo | NCT04685096 | | Cohort study; 89 healthy female aged 40-65 | External application: twice daily application of creme containing 2% NMN for 55 days | Wrinkles, eye bags, dark circles, relaxed features, skin texture, moisture, puffiness, brightness, youth, swelling, wrinkling, radiance and tone | Completed | United States and China | 2020 |
| the effects of a longevity supplement on aging and photoaging | NCT05262036 | | Open label, randomized, placebo-controlled study; 38 adults aged 35-70 years | Oral administration:mixture (powder) of NOVOS Core (12 ingredients) + NOVOS boost (NMN) daily for 6 months | Fine lines and wrinkles, facial redness, pigmentation and texture, skin hydration and elasticity, fasting lipids, hemoglobin A1c, ultra-sensitive CRP, blood pressure, mental health survey, subjective skin health survey, epigenetic signals | Not yet recruiting | United States | 2022 |
| Other | | | | | | | | |
| Trial Name | Registration number | | Design | Dose and duration | Indicators | Status | Location | Registration time |
| Effect of long-term oral administration of nicotinamide mononucleotide (NMN) on human health | UMIN000025739 | | Parallel, randomized study; 20 healthy adults aged 50-70 years | Oral administration: 100 mg/day and 200 mg/day for 24 weeks | Serum or plasma concentration of the following parameters:Thyroid-stimulating hormone (TSH), free triiodothyronine (Free T3), free thyroxine (Free T4), growth hormone (GH), prolactin, parathyroid hormone (PTH), dehydroepiandrosterone sulfate (DHEA-S), estradiol (E2), testosterone, calcitonin, adrenocorticotropic hormone (ACTH), arginine vasopressin (AVP), cortisol, aldosterone, ghrelin, inhibin, melatonin;  mitochondria activity in leucocyte, Sirt1 and Sirt2 gene expressions in leucocyte | Completed | Japan | 2017 |
| Exploratory study on the health promotion effect of intake NMN-containing supplement | UMIN000041677 | | Single arm, non-randomized study; 30 healthy male aged 40-65 years | Oral administration:1 capsule of NMN-containing supplement at breakfast daily for four weeks | Oxidative stress level, 8-OHdG in semen, quantity, count and concentration of Sperm, testosterone, creatine, spermine and zinc in semen, questionnaire (life style) | Completed | Japan | 2020 |
| A study evaluating various aging markers by taking NMN supplements | UMIN000045347 | | Open label, non-randomized, uncontrolled study; 15 healthy female aged 50-80 years | Oral administration:300 mg/day for 8 weeks | IGF-1, DHEA-s, free testosterone, cortisol, TSH, FT3, FT4, Insulin, NMN, NAD, Sirt1 mRNA, immunity judgment test, measurement of fluorescent AGEs (AGE reader), skin VAS questionnaire | Completed | Japan | 2021 |
| Effect of NMN supplementation on organ system biology (van) | NCT04571008 | | Randomized, placebo-controlled study; 56 healthy adults aged 45-75 years | Oral administration:300 mg/day for 16 weeks | Muscle insulin sensitivity, glucose tolerance | Recruiting | United States | 2020 |
| Effect of NMN on muscle recovery and physical capacity in healthy volunteers with moderate  Physical activity | NCT04664361 | | Double-blind, randomized, placebo-controlled study; 150 healthy male aged 20-49 years | Oral administration:250 mg/day and 500 mg/day for 38 days | Muscle recovery, physical capacity, cardiorespiratory recovery, blood lactate levels, perception of the intensity of post-exercise muscle pain (cramps), body composition, blood NAD^+^ levels | Recruiting | France | 2021 |
| Effect of oral NAD^+^ precursors administration on blood NAD^+^ concentration in healthy adults (NICO) | NCT05517122 | | Randomized, parallel-study; 68 healthy adults aged 18-50 years | Oral administration: NAM 500 mg/day, NR  1000 mg/day, NMN 1000 mg/day, for 14 days | NAD+ level in whole blood, for each NAD+ precursor | Recruiting | Switzerland | 2022 |
